# Left-handed cardiac looping by cell chirality is mediated by position-specific convergent extensions

**DOI:** 10.1016/j.bpj.2021.10.025

**Published:** 2021-10-23

**Authors:** Hisao Honda

**Affiliations:** 1 Division of Cell Physiology, Department of Physiology and Cell Biology, Graduate School of Medicine Kobe University, Kobe, Hyogo, Japan; 2 Laboratory for Morphogenetic Signaling, RIKEN Center for Biosystems Dynamics Research, Chuo-ku, Kobe, Hyogo, Japan

## Abstract

In the embryonic heart development of mammals and birds, a straight initial heart tube undergoes left-handed helical looping, which is a remarkable and puzzling event. We are interested in the mechanism of this chiral helical looping. Recently, observations were reported that myocardial cells in the embryonic chick heart show intrinsic chirality of rotation. The chirality of myocardial cells, via anisotropic polarization of Golgi inside the cells, leads to a left-right (LR) asymmetry of cell shape. On cell boundaries of LR asymmetric cells, phosphorylated myosin and N-cadherin are enriched. Such LR asymmetric cellular circumstances lead to a large-scale three-dimensional chiral structure, the left-handed helical loop. However, the physical mechanism of this looping is unclear. Computer simulations were performed using a cell-based three-dimensional mathematical model assuming an anterior-rightward-biased contractile force of the cell boundaries on the ventral surface of the heart (orientation of a clock hand pointing to 10 to 11 o’clock). An initially straight heart tube was successfully remodeled to the left-handed helical tube via frequent convergent extension (CE) of collective cells, which corresponds to the previously reported observations of chick heart development. Although we assumed that the biased boundary contractile force was uniform all over the ventral side, orientations of the CEs became position specific on the anterior, posterior, right, and left regions on the ventral tube. Such position-specific CEs produced the left-handed helical loop. In addition, our results suggest the loop formation process consists of two distinct phases of preparation and explicit looping. Intrinsic cell properties of chirality in this investigation were discussed relating to extrinsic factors investigated by other researches. Finally, because CE is generally exerted in the axial developmental process across different animal species, we discussed the contribution of CE to the chiral heart structure across species of chick, mouse, *Xenopus*, and zebrafish.

## Significance

In the embryonic heart development of mammals and birds, a straight initial heart tube undergoes left-handed helical looping, which is a puzzling event. Recent experimental observations have shown that an intrinsic cellular chirality caused the left-handed helical looping of the embryonic heart. However, the physical mechanism of this looping is unclear. We are interested in constructing a large-scale three-dimensional (3D) chiral structure of the helical heart tube based on the chirality at the cellular level. Using a cell-based 3D mathematical model, we succeeded in recapitulating the 3D dynamic formation of left-handed helical looping, in which asymmetric position-specific convergent extension (CE) of collective cells was key. The pathway from cell chirality to the asymmetric left-handed helical structure of the heart tube was elucidated.

## Introduction

In mammals and birds, embryonic development of the heart involves the conversion of a straight tubular structure into a three-dimensional (3D) left-handed helical loop. The structure and morphogenesis of heart looping have been investigated by many scientists (1, 2, 3, 4, 5, 6, 7). Heart looping is a mechanical event, and we were interested in the mechanism determining handedness. Cardiac looping in mammals and birds was first identified a century ago (8, 9, 10). At that time, Patten (10) proposed that looping results from a buckling mechanism in a tube elongating between fixed poles. Later, heart looping was observed to form through a combination of ventral bending and rightward rotation (11). Recently, helical looping definitively distinguished from simple bending was also reported to form through the combination of ventral bending and rightward displacement of the heart tube (12). Meanwhile, investigation to understand the cellular and subcellular mechanism of loop formation was performed. Local variation of the arrangement of actin bundles in the looping heart was first investigated in the chick embryonic heart (13,14), and later, the investigation was performed in 3D space (15). The role of actomyosin in the looping and bending of the heart tube was also investigated (6,14). Recently, Kidokoro et al. (16) observed actomyosin-based cell rearrangement and the resulting dynamic tissue reshaping in detail. They elucidated clearly that elaborated cell behavior, cell convergence, and tissue extension (CE) was exerted in the looping chick heart tube. Additionally, left-right (LR) asymmetry in the looping chick heart tube was analyzed at the cell and tissue levels using unique mathematical techniques (17). Recently, Ray et al. (18) confirmed a clockwise (CW) rotational chirality of cells in the developing myocardium. They sequentially examined the following: the chirality of the rotational behavior of myocardial cells, Golgi rightward polarization within cells (rightward means the orientation of a clock hand pointing to around 9 o’clock), the rightward-biased alignment of the cell boundary and LR asymmetry in cell shape, and enrichments of N-cadherin and phosphorylated myosin II (p-myoII) on the rightward-biased cell boundaries. Strong intensity p-myoII was predominantly aligned toward the anterior-rightward direction, which is expected to produce anisotropic force to lead to left-handed helical looping of the heart tube. These experimental observations were bases on which we constructed the heart model tube.

The purpose of this study is to elucidate the process of formation of the left-handed helical looping of the heart tube. Because the loop formation is a mechanical event of a cell assemblage being remodeled into a chiral structure, we used a cell-based 3D vertex dynamics in which we can introduce chiral properties of cells to the heart model tube. The cell-based 3D vertex dynamics is fundamentally different from other mathematical models that have been used for investigations of heart looping. Using this model we can examine process of loop formation in morphology of cell-level and remodeling of 3D structure of the heart tube.

First, we made a mathematically artificial tube whose surface consisting of polygons in cell-based 3D vertex dynamics system. We assumed anisotropic contractile force of the edges in the polygons (that is, edges of a certain direction have specifically contractile force). Computer simulations showed two cases: 1) a simple rotation of the tube surface around the sticking tube axis and 2) a large-scaled deformation of the tube itself producing a helical loop. We focused our interest on the second case to elucidate the looping mechanism of the heart tube. We made a straight tube similar to the embryonic initial heart tube under assumptions based on recently published observations. The mechanophysical mechanism of the formation of helical looping and how to determine the handedness of the helical looping were examined.

We found the process consisted of two distinct phases of implicit preparation of heart looping and explicit 3D remodeling of the looping heart tube. This investigation is based on the intrinsic properties of the myocardial cells, whereas other researchers have participated in the extrinsic factors of the heart looping. The relationship between contributions by the two factors was discussed. Finally, because CE is known to be generally exerted in the axial developmental process across different animal species (e.g., chick, mouse, *Xenopus*, and zebrafish), we discussed the formation of the chiral heart structure accompanied with CE across these animals.

## Materials and methods

Some of the methods used in this study were based on those presented previously (12), and are described in the Supporting materials and methods. The main features of the models are summarized here, and methods that are newly used (to our knowledge) in this study are described.

### Heart model tube

A mathematical tube that is used in computer simulations is referred to as a heart model tube, as distinguished from a real heart tube. The initial heart tube is a straight tube consisting of a cylindrical surface and two disks at the top and bottom extremities. Although the posterior end of the real heart tube is the bifurcation between the two atrial regions, we simplified it as a posterior pole. On the cylindrical surface, many polygons (452 polygons) are packed without gaps and overlaps. The polygons are approximations of myocardial cells in the heart tube and do not have thickness. Each cell is assumed to have its own polarity that is used as a reference line of anisotropic behaviors of cell edges and cell divisions (see Supporting materials and methods, Cell polarity). The model tube deforms and is remodeled during the computer simulations and its volume increases with time. Centers of the top and bottom disks (anterior and posterior poles, respectively) are fixed, although their peripheral vertices are movable.

### Calculation of vertex positions by equations of motion

To describe the shape of the heart model tube we require the *x*, *y*, and *z* coordinates of all vertices in the heart model tube and the neighboring relationships of vertices of the edges in the polygons. Changes in the neighboring relationships were performed using the elementary process of reconnecting the vertices (Fig. S1 *B*). Migrations of the vertices were calculated by the equations of motion (Eq. S1 in Cell-based 3D vertex dynamics in the Supporting materials and methods). The equation of motion involves potential *U*. The vertices move to decrease *U* (strictly, with no increase in *U*), as shown in Supporting materials and methods, Eq. S2.

The potential *U* in the equations of motion contains the terms for edge energy of the polygons, elastic surface energy of the polygons, elastic volume energy of the tube, elastic deviation energy of the vertices from the planes, and boundary restriction energy of the top and bottom of the model tube (Eq. S3 in the Supporting materials and methods). The ventral and dorsal sides of the heart model tube are distinct from each other. On the ventral side, we introduced the anisotropic contractile force of the edges in the cells.

### Anisotropic contractile force of edges

The potential *U* contains the term for the edge potential energy *U*_L_ = *σ*_L_ Σ_<*ij*>_ *w*_*ij*_*L*_*ij*_, as shown in Eq. S4 in the Supporting materials and methods, where *w*_*ij*_is the weight of edge *ij*. Anisotropic contractile force of edges is directed by the weight of edge that depends on orientation of edge. We can investigate anisotropic morphogenesis of the heart model tube using various values of the edge weight. In the cells on the ventral side, we introduce the anisotropic contractile force of edges; that is, we put *w*_*ij*_ equal to various *w*, depending on anisotropic angles. We do not consider edge contractile force on the dorsal side of the heart model tube.

### Determination of anisotropic contractile edges

**Figure 1:**
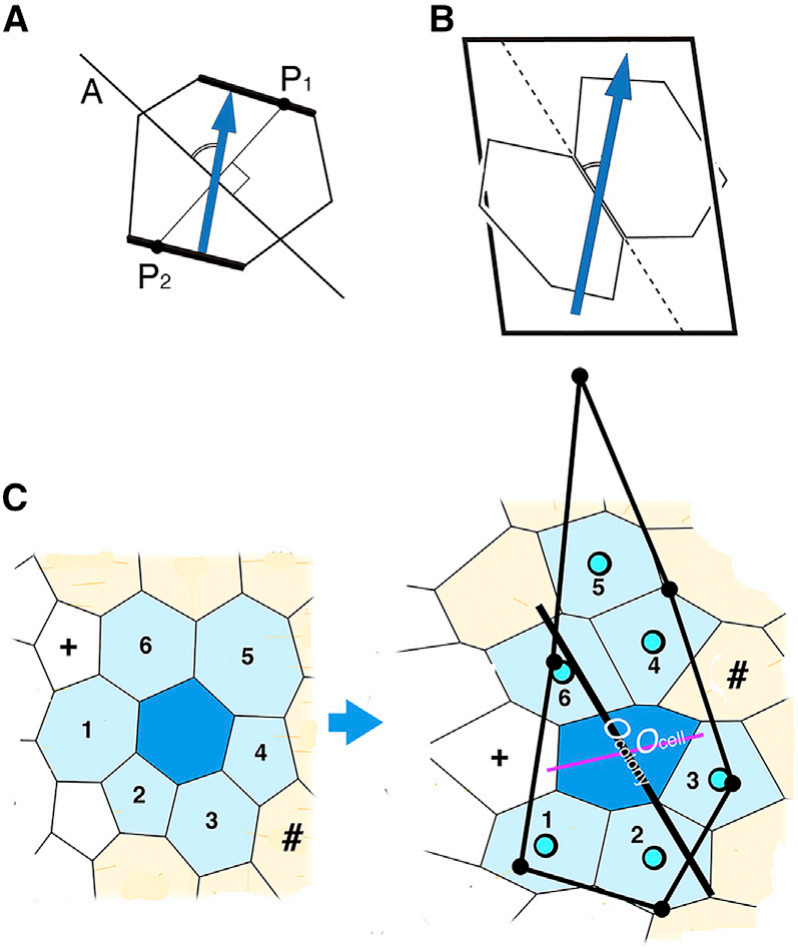
Figures for explanation of the computer simulation involving anisotropic properties. (*A*) Method to determine two specific edges in a polygon. An anisotropic angle line A is drawn, which forms the anisotropic angle (designated by *arc*) with respect to the polarity direction (*blue arrow*) where line A passes through the center of the polygon. Next, a line (*P*_*1*_*P*_*2*_) is drawn that is perpendicular to line A and includes the center of the polygon. Edges of a polygon that cross line P_1_P_2_ are designated as specific edges (*thick line*). Orientations of these two edges are closer to the anisotropic angle than other edges. (*B*) Method to measure an edge angle with respect to the polarity direction. An edge consists of two boundaries of neighboring polygons. Average orientation of polarities of the two polygons (*blue arrow*) is obtained from the direction cosines of polarities of the two polygons. An angle of the edge is the angle (designated by *arc*) between the edge direction and the average orientation of polarities (*blue arrow*). (*C*) Method to analyze change of colony shape during morphogenesis. Left and right patterns are at *t* = 0 and 75, respectively. A colony of closely neighboring cells (*faint blue cells* and *blue cell* at *t* = 0) is deformed to become a concave colony (*faint blue cells* and *blue cell* at *t* = 75). Colony *i* consists of cell *i* (*blue cell*) and *n*_*i*_ cells that surround cell *i* at *t* = 0. *n*_*i*_ is the number of surrounding cells at *t* = 0. The surrounding cells are designated by a faint (blue color) and referred to as cell *k* (*k* = 1, 2, 3, …, *n*_*i*_) throughout the analysis. Their central points at *t* = 75 are designated by small blue circles. Note that cells designated by the plus sign (+) and the symbol (#) do not belong to cell *k,* although they are neighbors of cell *i* at *t* = 75, because the cells designated by the plus sign (+) and symbol (#) were not the neighbors of cell *i* at *t* = 0. The central points of cell *k* (*k* = 1, 2, 3, …, *n*_*i*_) at *t* = 75 are normalized (*solid black circles*) as follows: polygon *i*, whose vertices are the central points of colony member *k,* was normalized using a ratio of distances *r*_*k*_*/r*_*0k*_, where *r*_*0k*_ and *r*_*k*_ are distances of cell *k* from cell *i* at *t* = 0 and *t* = 75, respectively. Positions of central points of the normalized colony polygon were (*x*_*k*_*′*, *y*_*k*_*′*), where *x*_*k*_*′* = *x*_*i*_ + (*x*_*k*_ − *x*_*i*_) *r*_*k*_/*r*_*0k*_ and *y*_*k*_*′* = *y*_*i*_ + (*y*_*k*_ − *y*_*i*_) *r*_*k*_/*r*_*0k*_ (*black solid circles* in Fig.1*C*, on the *right*). This normalization enabled us to obtain net changes of colony shape anisotropies at *t* = 75, regardless of the colony shape at *t* = 0. A polygon whose vertices are the normalized central points (*solid black segment line*) was analyzed using the approximation method of a momental ellipse. Orientations of red and black segment lines represent cell orientation (*O*_cell_) and colony orientation (*O*_colony_), respectively. Lengths of segment lines represent cell shape anisotropy (*A*_cell_) and colony shape anisotropy (*A*_colony_), respectively. To see this figure in color, go online.

It was necessary to determine the strong contractile edges, depending on their orientation. However, we do not to consider that an edge has the ability to measure its orientation in 3D space and judge whether it has strong contractile properties or not. Rather, a cell determines which edges in the cell should have a strongly contractile force, referring to its polarity. Therefore, we determined particular edges whose orientations were close to an anisotropic angle, as shown in Fig. 1 *A*. Particular edges are designated by a thick line.

### Strength of contractile force of each edge

**Figure 2:**
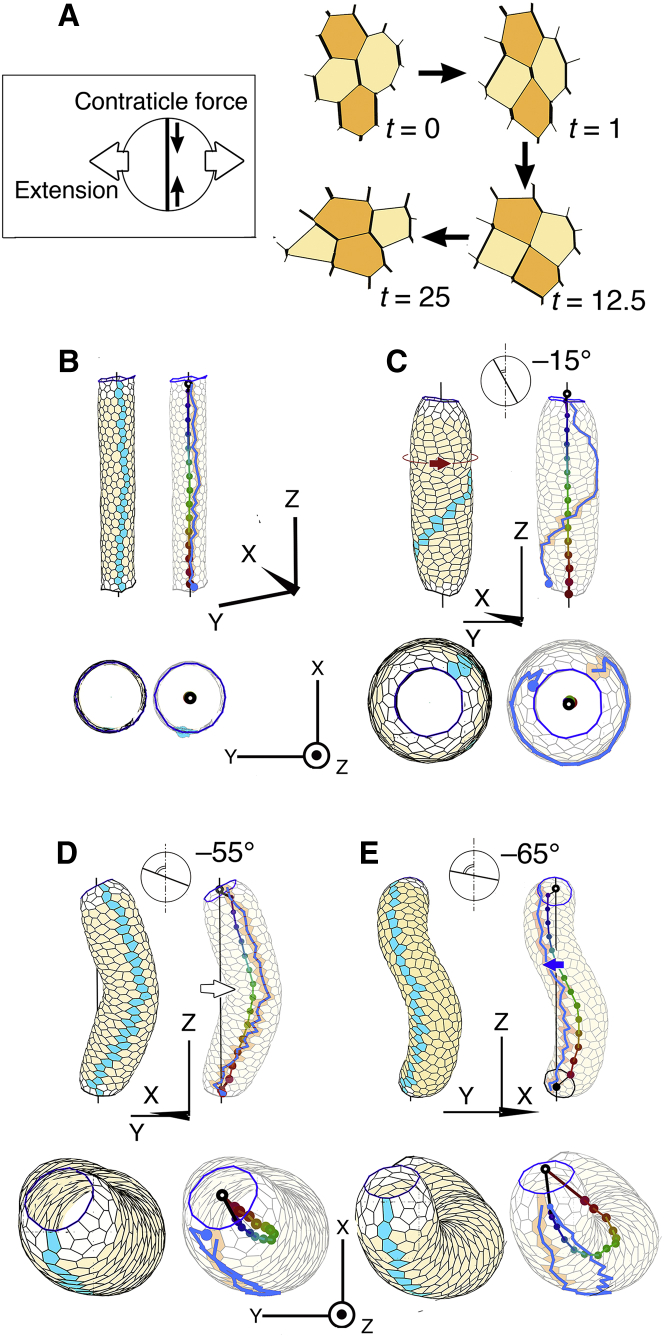
Shape changes of an artificial tube by anisotropic contractile force of edges. (*A*) Rearrangement of four polygons by anisotropic contractile force of edges generates collective motion of convergent extension (CE). Patterns at *t* = 0, 1, 12.5, and 25 are shown. Vertical edges have strong contractile force (*thick line*). Two vertically separated polygons (*orange color*at *t* = 0) converge to be adjacent with each other, and a pattern of four polygons extends horizontally (*t* = 25). (*Inset*) Horizontal extension of distance between two neighboring cells by vertically contractile force of edges. (*B*−*E*) Side view (*top*) and its horizontal projection (*bottom*). Blue line designates array of blue polygons. Colored chain designates central line of the tube. Translucent tube image is superimposed. (*B*) Initial tube. A cylinder, whose surface is covered by polygons, is shown. An array of vertically arranged polygons on the tube surface is designated by gray polygons. *t* = 0. (*C*) Right-handed screw rotation of polygons on the tube surface, just like a barber’s pole, by the anisotropic contractile force of edges (anisotropic angle = −15°). The line of the blue polygons rotated∼270° around the tube axis of the colored chain. *t* = 150. (*D*) Hairpin-like bending of the tube by the anisotropic contractile force of edges (−55°). Blue line and colored chain are almost parallel to each other. *t* = 150. (*E*) Left-handed helical looping of the tube by the anisotropic contractile force of edges (−65°). Blue line and colored chain are almost parallel to each other. *t* = 150. To see this figure in color, go online.

**Figure 3:**
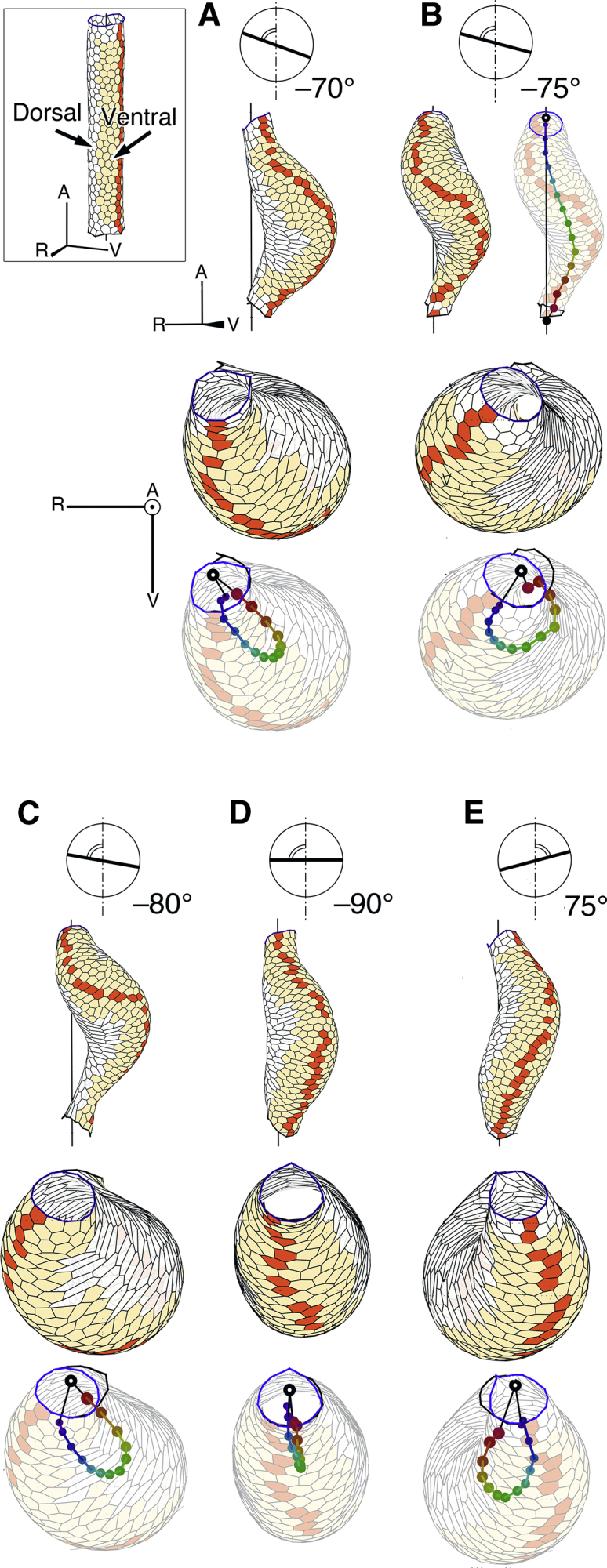
Shape changes of the heart model tube, depending on anisotropic angles of strong contractile edges. (*Inset*) An initial heart model tube consisting of the ventral and dorsal sides is shown. Orange cells in the ventral side perform shape changes by the anisotropic contractile force of edges. *t* = 0. (*red polygons*) A vertically arranged cell array at the ventral-most on the heart model tube surface. (*A*−*E*) Shapes of the model tube with anisotropic edge angles = −70, −75, −80, −90, and +75°. *t* = 150. Edges of angles close to the anisotropic angle have strong contractile force. From top to bottom row, circular presentation of anisotropic edge angle (*clockwise* is positive), side and top views of the model tube, and presentation of color chain pattern that show the central line of the model tube, where translucent tube images are superimposed. A, V, and R represent the anterior, ventral, and right directions of the initial model tube, respectively. To see this figure in color, go online.

An edge in polygonal patterns consists of two boundaries of two neighboring cells (Fig. 1 *B*). For cells of the artificial tube (Fig. 2) and on the ventral side of the heart model tube (e.g., Fig. 3), the strength of the edge contractile force was considered to be the sum of contributions from the two cell boundaries of neighboring cells. For example, edge <*ij*> is a boundary between cells A and B and has two contributions from cell A and cell B, *w*_*ij*_^A^ and *w*_*ij*_^B^. The strength of edge contractile force of the edge <*ij*> is *w′*_*ij*_ = *w*_*ij*_^A^ + *w*_*ij*_^B^ (see Eq. S4 in the Supporting materials and methods). Usually, we use *w* as the weight of the strength of a strong contractile force of an edge (i.e., contribution from one cell). When the weight of the strong contractile edges is *w* = 2.7 and the weight of the other edges is 1.0, the edges on the ventral side of the heart tube have three strength levels; that is, *w′* can be 2 (= 1 + 1), 3.7 (= 1 + 2.7), or 5.4 (= 2.7 + 2.7).

### Measurement of edge angle with the polarity direction

To analyze results of computer simulations we have to measure edge angles. An edge is defined by two neighboring polygons (Fig. 1 *B*). Average orientation of polarities of the two polygons (*blue arrow*) is obtained from direction cosines of polarities of the two polygons. An angle of the edge is the angle (designated by *the arc*) between the edge direction and the average orientation of polarities (*blue arrow*).

### Shape anisotropy and orientation of cells

Each polygonal cell area *S* was measured at *t* = 80. A polygonal cell was approximated by a momental ellipse (an ellipse of inertia). The principal axis and the second axis of the ellipse were *a* and *b*, respectively. Cell orientation, *O*_cell_, was defined as an angle of the principal axis of the ellipse from the anterior-posterior axis. Cell anisotropy was defined as *A*_cell_ = (*a* − *b*)/*a*.

### Shape anisotropy and orientation of colonies around a cell

We considered a colony around cell *i* **(**Fig. 1 *C*, *t* = 0**)**. Colony members *k* surround cell *i* at *t* = 0 (*faint blue polygons* in Fig. 1 *C*). *k* = 1,… *n*_*i*_, where *n*_*i*_ is number of cells surrounding cell *i* at *t* = 0. We quantified the colony shape at *t* = 80 as follows. At first, polygon *i*, whose vertices are the central points of colony member *k*, was normalized as described in the legend of Fig. 1 *C*. The normalization enabled us to obtain net changes of colony shape anisotropies, regardless of the colony shape. The normalized colony polygon was approximated by a momental ellipse. The principal axis and the second axis of the ellipse were *a′* and *b′*, respectively. Colony orientation, *O*_colony_, was defined as an angle of the principal axis *a′* of the ellipse from the anterior-posterior axis. Colony anisotropy was defined as *A*_colony_ = (*a′* − *b′*)/*a′*. Orientation and length of solid black segment line are *O*_colony_ and relative scale of *A*_colony_, respectively (Fig. 1 *C*, *right*). Note in Fig. 1 *C* that, although the blue cell is flattened horizontally (*O*_cell_; *red line segment*), the colony involving *n*_*i*_ cells *k* is elongated longitudinally (*O*_colony_; *black line segment*). In comparison to *O*_cell_ and *A*_cell_, *O*_colony_ and *A*_colony_ are higher-sensitive indicators because the cells migrate independently and dynamically through repetition of cell intercalation.

## Results and discussion

### The first-round computer simulation

We started with a sheet consisting of polygons and first looked at four neighboring polygons in this sheet (Fig. 2 *A*). Contractile force of specific edges was assumed; that is, that edges whose directions are close to the vertical direction have strong contractile force (anisotropic contractile force) and the change in the polygonal pattern was examined by using a mathematical model system (see Cell-based 3D vertex dynamics in the Supporting materials and methods). As shown in Fig. 2 *A*, several edges of the four polygons expressed strong vertical contractile force (thick solid line) and the edges were rearranged with each other exchanging connections at vertices. This is a topological pattern transformation. Two neighboring polygons (*faint orange color*) were intercalated by two dark orange-colored polygons as shown in Fig. 2 *A* (*t* = 0–25). A pattern of the four polygons changed from a vertical to horizontally elongated shape (*t* = 25). Therefore, the anisotropic contractile force of the edges enables a pattern of polygons to be expanded perpendicularly to the direction of contractile force (Fig. 2 *A* inset). This is a collective motion of CE. CE of tissues was first identified in the neural plate (19) and has been found in many other tissues. CE caused by the anisotropic contractile force of edges was demonstrated at the cellular level by computer simulation of the mathematical model (20,21).

We applied the above-mentioned anisotropic contractile force to a mathematically artificial model of a straight tube consisting of many polygons (Fig. 2 *B*). Several simulations were performed with various anisotropic angles. At first, we applied an anisotropic angle of −15° (Fig. 2 *C*), where edges whose direction was close to −15° expressed strong contractile force. The direction of −15° is the orientation of a short clock hand pointing to 11:30 o’clock. The polygons migrated perpendicularly to the anisotropic angle and to form a right-handed screw pattern on the surface of the tube (Fig. 2 *C*, *red arrow*). Indeed, the array of blue polygons that had been vertical in Fig. 2 *B* rotated to form a right-handed screw, just as a barber’s pole would, as shown in Fig. 2 *C*. When we changed the anisotropic angle from −15° to −55°, the array of blue polygons rotated backward. The array of blue polygons did not rotate on the tube surface at the anisotropic angle of −55°. Instead, the tube itself mechanically 3D deformed to a bent shape, as shown in Fig. 2 *D* (*white arrow*). The array of blue polygons followed the bending of the tube. The bottom figure shows that the central line of the tube, shown as a colored chain, forms a hairpin-like shape (see Centers of sliced model tubes in the Supporting materials and methods). The hairpin-like shape of the colored central line indicates that the tube is bent. The bend looks like buckling of the tube. The array of blue polygons runs parallel with the hairpin-like shape of central line of the tube. Moreover, when we changed the anisotropic angle to −65°, the tube not only became bent but also deformed its entire shape to be a helical loop (Fig. 2 *E*, *blue arrow*). The array of blue polygons on the tube surface followed the helical looping of the central line. It should be noted that the helical tube formed a left-handed helix with the anisotropic angle to −65°, whereas the spiral rotation of the tube surface with the anisotropic angle to −15° was right-handed (Fig. 2 *C*). We understood these results when we considered the horizontal and vertical components of the extension force separately as follows. As the absolute anisotropic angle increased (from 15 to 65°), the horizontal component of the extension force decreased, and surface rotation stopped. Instead, the vertical component force increased so that the tube itself deformed to be bent. Taken together, the anisotropic angles of −55° is a critical angle for the deforming heart model tube. When absolute anisotropic angle is less than 55°, the array of blue polygons on the tube rotates around the tube axis. When it is more than 55°, the array is fixed to the surface of tube and moves in parallel with the twisting tube. In conclusion, the anisotropic contractile force of the edges of the polygons on the tube surface causes either 1) spiral rotation of the polygons on the tube surface (rotation of a barber’s pole) or 2) 3D deformation of the entire tube shape. When the cell arrays are difficult to rotate because of weak component force of the horizontal direction, the tube changes its 3D shape to become a helical loop. This result shows that we have a powerful mathematical cell model, by which we can construct 3D tissues that are remodeled in large-scale. Also, we already know that the anisotropic forces cause rotation of the tissue surface in the hindgut and genitalia of *Drosophila* (22,23).

### Computer simulations of the initial heart tube

The above-mentioned computer simulation showed that the anisotropic contractile force of the edges of polygons causes an artificial tube to loop helically under certain conditions. This result encouraged us to elucidate a physical mechanism underlying the helical looping of the real heart tube. We considered a model tube for the initial embryonic heart (Fig. 3, *inset*). The model tube consists of the ventral and dorsal sides, in which cells in the dorsal side do not have any anisotropic properties.

We performed computer simulations with various anisotropic angles of cells in the ventral side (Fig. 3). Strong contractile force of edges whose angles were close to the anisotropic angle of −70, −75, and −80° produced left-handed helical looping (Fig. 3, *A*–*C*). These patterns were more or less the same. The results show degree of robustness of the anisotropic angle. When a horizontal edge expressed strong contractile force (anisotropic angle = −90°), the model tube simply bent, showing a hairpin pattern (Fig. 3 *D*). When we used an inverse angle (anisotropic angle = +75°), we obtained an expected inverse (right-handed) helix loop (Fig. 3 *E*).

Next, we examined the effect of differential strength of contractile force of edges in the ventral cells (Fig. S2). *w* is the relative weight of the strength of contractile force of a specific cell edge (see Strength of contractile force of each edge in Materials and methods). To edges whose angles were close to an indicated anisotropic angle, we used a large *w* (e.g., *w* = 3), whereas the *w* of the other cell edges was set to 1. For examination of the effect of differential contractile force of edges, we fixed the anisotropic angle at −75°. When we did not consider specific cell edges (i.e., all cell edges had *w* = 1), the model tube did not loop but bent instead (Fig. S2 *A*). When we used *w* = 2.7, we obtained a helical loop as shown in Fig. S2 *C*. Similar results were obtained when *w* = 2.4 and 3.0 (Fig. S2, *B* and *D*). These simulations show the degree of robustness of the strength of contractile force of the specific edge. A course of formation of the helical loop is shown in Fig. S3. Before notable changes to the shape of the model tube, the polygonal pattern of cells on the tube surface changed from a hexagonal pattern, resembling a pattern in bee’s nest (*t* = 0), to a horizontal array of polygons (*t* = 20), as shown in the inset in Fig. S3. The vertical array of the ventral-most cells (*red cells*) was intercalated by neighboring cells. The model tube began to loop at *t* = 50 and became a distinct left-handed helical loop (*t* = 110 and 150). Horizontally projected figures (*bottom*, *t* = 110 and 150) obviously showed the left-handed helical loop. Patterns of *t* = 110 and 150 seem to correspond to the embryonic chick heart tube just before HH12 (Fig. 1 G in (11)).

### Distribution of edges with anisotropically contractile force

**Figure 4:**
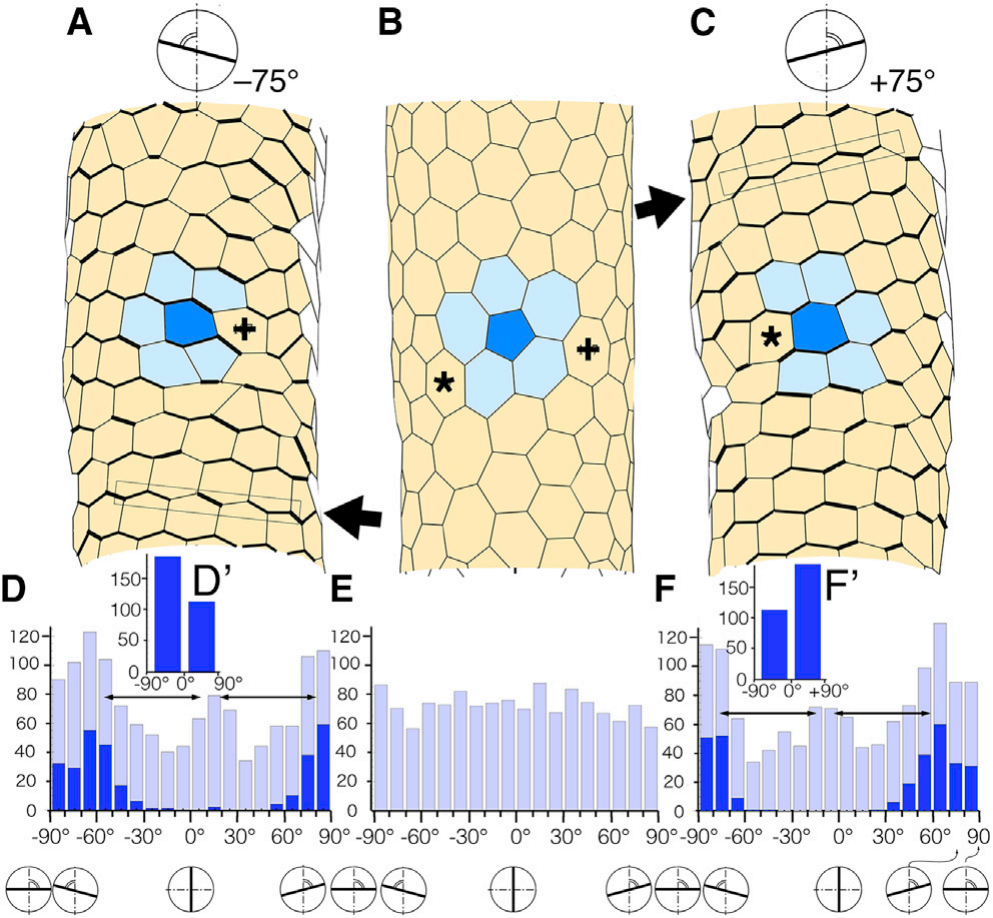
Confirmation of distribution of anisotropic edges with strong contractile force in the computer simulations. We have assumed that edges whose orientation was close to the anisotropic angle have strong contractile force. Here, the edges that have strong contractile force were examined with the results of the early stage of computer simulations (*t* = 12.5). (*A* and *C*) Partially enlarged areas of heart model tubes with anisotropic angles −75 and +75° are shown (*t* = 12.5). Horizontal cell arrays are indicated by narrow rectangles, which are slightly tilted to the CW or CCW direction (*arrow*). (*B*) Control. The partially enlarged area of model tube of *t* = 0 is shown. The dark blue polygon (*center* of figures) has five neighbors at *t* = 0. The neighbors increased by one (cell marked with *plus sign* (*+*) or *asterisk* (^∗^)) at *t* = 12.5 (*A* and *C*). Orange polygons are cells of the ventral half of the initial model tube. (*D*, *D′*, *F*, and *F′*) Analysis of orientations of edges with strongly contractile force in the model tubes at *t* = 12.5. Results of computer simulations with the assumption of anisotropic angles −75° (*D* and *D′*) and +75° (*F* and *F′*) are shown. (*E*) Analysis of orientations of edges of polygons in the model tubes (*gray bar*, *t* = 0). Distributions of edge angle are shown (*gray bar*; *D*, *E* and *F*). Angle distribution that is approximately even at t = 0 (*E*) changed to have three peaks with a 60° interval at *t* = 12.5 (*horizontal arrows with double arrowhead* in *D* and *F*). Numbers of strong contractile edges are analyzed (*dark blue bar*). Comparison between the total numbers of edges with a negative angle (−90 to 0°) and a positive angle (0 to +90°) is performed (186 and 113 in *D′*; 114 and 189 in *F′*). Statistics for (*D′*) and (*F′*) are as follows: the null hypothesis that the orientations of edges with strongly contractile force ([−90 to 0°] or [0 to +90°]) and the anisotropic angles (−75 or +75°) in the assumption are independent was rejected (*p* < 0.001 by *χ*2 test, test for independence); degree of freedom 1; *χ*2 statistics = 36.38; *χ*2 statistics_*p* = 0.001_ = 10.83). In conclusion, when we assumed that anisotropic angle of edge is −75° and analyzed numbers of the edges with strong contractile force, edges with negative orientation (−90 to 0°) are larger than those with positive orientation (0–90°), as shown in (*D′*). When we assumed that anisotropic angle of edge is +75°, the opposite was shown in (*F′*). To see this figure in color, go online.

Ray et al. (18) observed p-myoII on cell boundaries in the ventral myocardium at HH9 and reported that the intensity of p-myoII on cell boundaries of angles between −90° and 0° was higher than that of angles between 0° and 90°. Here, to obtain the result of Fig. S3 (*t* = 150), we used the weight of anisotropic force strength of edge *w* = 2.7. We confirmed that the angle distribution under the assumption of *w* = 2.7 was compatible with the observation of Ray et al. (18) as follows. The model tube at *t* = 12.5 was almost straight, as shown in Fig. S3 (*t* = 12.5). An enlarged polygonal pattern is shown in Fig. 4 *A*, where edges with strong contractile force (large *w*-value) are represented by a thick black line. We measured the angles of all edges of the polygons in the ventral side in the polygonal pattern of *t* = 0 (Fig. 4 *B*) and *t* = 12.5 (Fig. 4 *A*) using the method described in Measurement of edge angle with the polarity direction in Materials and methods. The results of these analyses are shown in Fig. 4, *E* and *D*. The angle distribution that had been roughly even at *t* = 0 (Fig. 4 *E*) came to have three peaks, with an∼60° interval at *t* = 12.5 (Fig. 4 *D*). The three peaks remind us of a hexagonal pattern resembling the pattern in a bee’s nest. Angle distribution of the edges with strong contractile force is shown in Fig. 4 *D* (*dark gray bar*). The total number of edges of strong contractile force with a negative angle (−90° to 0°) and a positive angle (0°–90°) are shown in Fig. 4 D′. The number of edges with negative angles was certainly larger than that of edges with positive angles, which corresponded with the observation of Ray et al. (18). We performed a similar examination in the results of other computer simulations with an anisotropic angle of +75°, as shown in Fig. 4 *C*. The angle distribution of edges is shown in Fig. 4, F and F′. Differences between the results with the anisotropic angle −75° (Fig. 4 D′) and +75° (Fig. 4 F′) were significant, as described in the legend of Fig. 4. Thus, we confirmed that the method of Determination of anisotropic contractile edges in Materials and methods was appropriate to computer simulations of the embryonic heart tube.

### Embryonic chick heart tubes consist of heterogeneously chiral cardiomyocytes

Ray et al. (18) demonstrated that chick myocardial cells were intrinsically chiral and exhibit dominant CW rotation in vitro, and myocardial cell chirality in the heart tube controls the directionality of cardiac looping. However, the chirality of myocardial cells was not homogeneous. Myocardial cells in the rotation system showed a 62.5–74.1% CW rotation and a 37.5–25.9% counter clock-wise (CCW) rotation of cells (Table S1), which suggests a left-handed helical heart consisting of cells not only with edges of the rightward strong contractile force but also with edges of the opposite chiral type.

We thus examined the robustness of the handedness of helical looping in the model tubes. Using a series of random numbers, we made nine heart model tubes consisting of various ratios of cell types (0.1–0.9), where type ratio is a ratio of cell number of −75° type to the total cell numbers of both types. We examined the handedness of the resultant twisted model tube. Results are shown in Fig. S4 (*top*). Color chains that represent the central lines of tubes were projected on the horizontal plane. The patterns of projected color chains indicate whether or not model tubes are in a helical loop conformation. These conformations were left-handed helix, hairpin-like bent, and right-handed helix, sequentially from left to right in Fig. S4 (*top*). Changing between left- and right-handed helices took place around a ratio of 0.4 and 0.5. We performed four further computer simulations with model tubes made by another series of random numbers. All results were presented by line chart. The green line was the result of the first series of random numbers. Four other similar computer simulations are represented with black lines. The data were analyzed using statistics of *t*-test as shown in the legend of Fig. S4. Results of our computer simulations of the heart model tube are consistent with the observation of Ray et al.(18).

### Cell rearrangement took place frequently in the early stage of the helical loop formation

We showed that CE leads to a left-handed helical loop. To elucidate the mechanophysical mechanism of the formation of the left-handed helical loop, we examined the process in detail using computer simulations. Fig. S5 *A* shows the frequency of the rearrangement of four polygons during the helical loop formation. The rearrangement was particularly concentrated until *t* = 10, then ceased. The rearrangement after *t* = 10 appeared to be unnecessary for the loop formation. Indeed, we performed another computer simulation in which the rearrangement did not take place after *t* = 10, and a similar left-handed helical loop was obtained as shown in Fig. S5 *A*. We obtained a similar result by computer simulation with anisotropic angle = +75°, in which we obtained the right-handed helical loop (Fig. S5 *B*). The results are discussed with the process of the helical looping in General discussion.

A comparison between the early stage of cell patterns (*t* = 12.5) with anisotropic angles = −75° and +75° showed a delicate but precise difference (Fig. 4, *A* and *C*). Some of the horizontal cell arrays are observed in Fig. 4 *A* (designated by a *narrow rectangle and arrow*) and the narrow rectangle is slightly tilted to the CW direction. We also observed an opposite tilt of the narrow rectangle in the model tube with a +75° anisotropic angle (Fig. 4 *C*, *arrow*). As already mentioned, we examined the angle distribution of the edges with strong contractile force (Fig. 4 *D*, *dark blue bar*). The number of edges with an angle between −90° and 0° was certainly larger than that between 0° and 90° (Fig. 4 D′). The opposite result was obtained in the model tube with a +75° anisotropic angle (Fig. 4 F′). In addition, we found interesting changes in cell patterns. A blue cell in Fig. 4 *B* (*t* = 0) had five surrounding neighbor cells that enclosed the blue cell. Afterward, there were six surrounding neighbor cells for the blue cell. The manner of addition of one cell was different between the model tubes of −75° and +75° anisotropic angles (Fig. 4, *A* and *C*). A new surrounding cell in Fig. 4 *A* was from the right side (cell designated by a *plus sign* (+)), whereas a new surrounding cell in Fig. 4 *C* was from the left side (cell designated by an *asterisk* (^∗^)). These LR asymmetric behaviors of cells were suggested to be the results of the anisotropic cell properties and the causes of the left- and right-handed helical looping. Cell rearrangements in the heart model tube were almost settled until reaching *t* = 12.5. Such cell rearrangement did not notably change the whole view of the tube shape (*t* = 12.5–20; see Fig. S3). Thereafter the cells were elongated and enlarged without cell rearrangement, and a large-scale morphogenesis of helical looping took place. Together, the collective motion of CE and the cell rearrangement did not have an explicit effect on helical looping immediately but provided potential abilities of the formation of large-scaled helical looping.

### Position-specific deformation of cell colonies in the process of the helix loop formation

**Figure 5:**
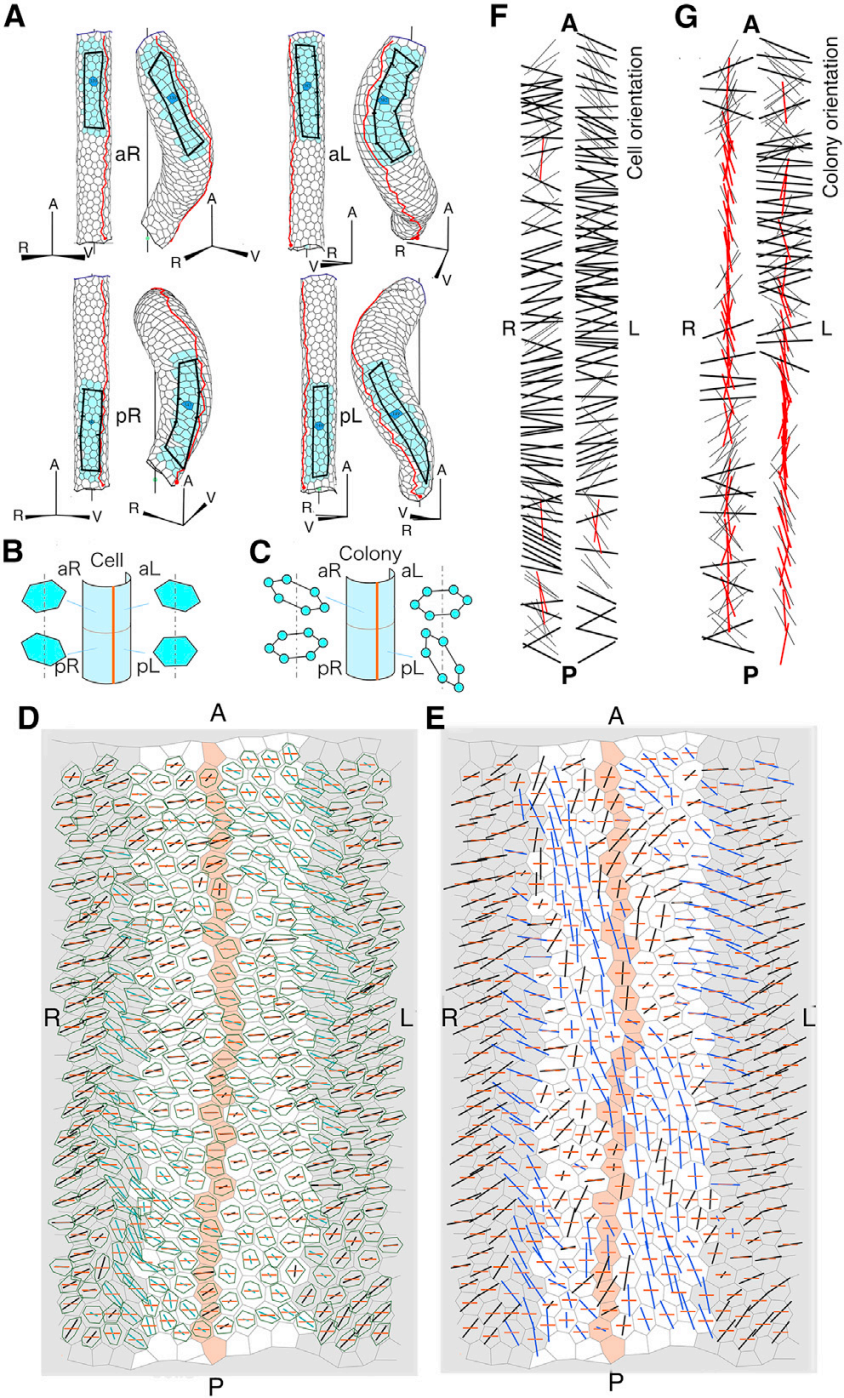
Analysis of cell/colony orientations and cell/colony shape anisotropies in the heart model tube with −75° anisotropic angle. (*A*) Regional changes of cell patterns during the helical loop formation of the heart model tube with a −75° anisotropic angle. Cell patterns of the aR, aL, pR, and pL regions are shown. Right and left in each figure are heart model tubes at *t* = 0 and 80, respectively. Cells in each region are faintly colored. Flames of rectangles are drawn for recognition of pattern changes. Each view direction of the four figures is different. The central cell of each region is dark-colored, the normal of which is a view line that is perpendicular to the page. A, anterior; R, right; V, ventral; aL, anterior left; aR, anterior right; pL, posterior left; pR, posterior right. (*B* and *C*) Schematic representation of averaged orientation and shape anisotropy (*O*_cell_, *A*_cell_) of cells (*B*) and averaged orientation and anisotropy (*O*_colony_, *A*_colony_) of colonies (*C*) using hexagons, respectively. Averaged orientation and shape anisotropy are expressed by the main axis of the hexagon and the shape of the hexagon, respectively. (*D*) Cell orientation (*O*_cell_) and cell shape anisotropy (*A*_cell_) of a heart tube with a −75° anisotropic angle. Direction and length of line segments indicate the orientation of *O*_cell_ and relative strength of *A*_cell_, respectively. Black and blue lines represent angles of line segment from the vertical direction that are CW (positive angle) and CCW (negative angle), respectively. Red line indicates the horizontal direction. Green polygon shows a polygonal cell at *t* = 80 in relative scale. These are plotted on a plane, which is an unfolded sheet of the lateral surface of the initial heart model tube of *t* = 0. Orange cell array represents cells that were at the ventral-most position in the heart tube. Gray zone is dorsal region in the heart model tube. P, posterior, R, right. (*E*) Colony orientation (*O*_colony_) and colony shape anisotropy (*A*_colony_) of a heart tube with a −75° anisotropic angle. Direction and length of line segments indicate the direction of *O*_colony_ and relative strength of *A*_colony_, respectively. For other notes, see legend of (*D*). (*F* and *G*) Superimposed presentation of line segments of cell orientation and colony orientation. Line segments of cell orientation in the left and right regions of the heart model tube are arranged on each column (*F*). Line segments of colony orientation are arranged similarly (*G*). Lines whose directions are closed to the horizontal line (i.e., absolute angle from the vertical direction, |*O*_cell_| > 60°, |*O*_colony_| > 60°) are drawn with thick black lines. Lines whose directions are closed to the vertical line (i.e., absolute angle from the vertical direction, |*O*_cell_| < 15°, |*O*_colony_| < 15°) are drawn with red lines. To see this figure in color, go online.

To examine the relationship between cell deformation in local regions and entire helical looping during left-handed helical loop formation, we divided and analyzed the ventral side of the model tube in four regions: anterior left (aL), anterior right (aR), posterior left (pL), and posterior right (pR). We did not analyze the dorsal cells because we assumed that the dorsal cells have no anisotropic properties in the computer simulations. During the process of *t* = 0–80, Fig. 5 *A* shows that the aR region extended longitudinally and the aL region extended with curving, which was compatible with the observations of the chick embryonic heart by Kawahira et al. (17). In the posterior region, the results were the opposite. The pR region extended with curving and the pL region extended longitudinally. Furthermore, we examined behaviors of each cell and its neighbors in detail. We measured the cell area (*S*), cell shape anisotropy (*A*_cell_ = (*a* − *b*)/*a*, where *a* and *b* are the longest and the shortest axes of approximated ellipses, respectively), and cell orientation (*O*_cell_; orientation of the longest axis *a*) (see Materials and methods). To examine the deformation of the neighborhood of each cell, we observed a colony of surrounding cells around each cell. We measured colony shape anisotropy (*A*_colony_ = (*a′* – *b′*)/*a′*, where *a′* and *b′* are the longest and the shortest axes of approximated ellipses, respectively) and colony orientation (*O*_colony_; orientation of the longest axis of colony *a′*). Results of the measurement are shown in Fig. 5, *D* and *E*, in which the lateral surface of the heart model tube was unfolded in a plane. Each measured value of cells was plotted on the plane by a line segment whose orientation was *O*_cell_ and whose length was relative *A*_cell_ (Fig. 5 *D*). Cell shapes at *t* = 80 were plotted in green. Each measured value of colony was also plotted in the other plane by a line segment whose orientation was *O*_colony_ and whose length was relative *A*_colony_ (Fig. 5 *E*).

Comparison between Fig. 5, *D* and *E*, colony orientation and anisotropy (*O*_colony_ and *A*_colony_) differed significantly from cell orientation and anisotropy (*O*_cell_ and *A*_cell_). *O*_colony_ and *A*_colony_ are higher-sensitive indicators of shape change than *O*_cell_ and *A*_cell_ because the cells in a colony migrate individually and dynamically through repetition of cell intercalation. Statistics of these measurements in each region are presented in Fig. S7 *A* and Table S2 *A*. In the anterior and posterior regions, we performed *t*-tests of these five terms and confirmed that the difference of these terms between the left and right sides were significant (Table S2 *A*). The result is shown schematically in Fig. 5, *B* and *C*. When we examined the difference between the left and right side in the total data of the anterior and posterior regions, the difference was not significant (Fig. S7 *A*; Table S2 *A*). The result was in agreement with the observations by Kawahira et al. (17). We also performed a similar examination of the right-handed helical loop (with a +75° anisotropic angle) and obtained similar results, as shown in Fig. S6, Fig. S7 *B*, and Table S2 *B*. The simulation with a +75° anisotropic angle was used to show that the bias of LR asymmetry of the initial model tube was negligible. Because the initial model tube had been made using random numbers (as described in Construction of the initial model tube in the Supporting materials and methods), we had been concerned about the bias of the LR symmetry of the initial model tube. We confirmed that the bias of the initial model tube was so small that it did not disturb the determination of the handedness of helical looping.

### Mechanophysical mechanism that determines the handedness of the helical loop

Which term made the largest contribution to determination of the handedness of the helical looping among the above-mentioned changes of cells and colonies? To answer this question, we superimposed line segments expressing cell orientations (*O*_cell_) of the left side on the left column and line segments expressing cell orientations of the right side on the right column (Fig. 5 *F*). We also superimposed line segments of colony orientations (*O*_colony_) of the left and right regions in a similar way (Fig. 5 *G*). The superimposed line segments of colony orientations should be noteworthy. The distribution of line segments of colony orientations close to the horizontal direction was outstandingly different between the left and right sides. There were many roughly horizontal line segments in the anterior half of the left column, whereas there were few in the anterior half of the right column. The LR asymmetric distribution was opposite in the posterior half. Longitudinal expansion close to the vertical direction was also remarkable in the anterior half of the right column and the posterior half of the left column. These results show that the colony orientations (*O*_colony_) are considered to be the primary factor in the determination of the handedness of the helix. We also performed a similar analysis of the right-handed helical loop with a +75° anisotropic angle (Fig. S6; Table S2 *B*). The result was confirmed to be inverse asymmetric; that is, the left-right (LR) bias of the initial heart model tube was negligible. On the other hand, the difference of the cell orientation (*O*_cell_) between the left and right sides was not so clear, as shown in Fig. 5 *F*. Cell shapes seemed to not be deeply correlated with the handedness of helical looping.

### General discussion

#### Distinctive feature of the cell-based vertex dynamics model

In this study, we succeeded in making a connection between the chirality of myocardial cells and the handedness of the helical heart tube via anisotropic cell behavior. To investigate the physical mechanism producing the handedness, mathematical models were indispensable. Previously, a few mathematical models had been used in the investigation of looping of the heart tube. Shi et al. (24) constructed a finite element analysis model and recapitulated bending and torsion of the heart tube. Computer simulations using another finite element analysis model were performed and demonstrated a recapitulation of large-scale dynamic heart looping (7). We note the advantage of cell-based vertex dynamics over the finite element analysis model. In the finite element analysis, the heart tube was assumed to be a sheet of continuous material rather than an assembly of discrete cells. In the cell vertex dynamics used in our simulation, it was instead possible to assume cell polarity, anisotropic edge properties, chiral properties of individual cells, and orientation of cell division in individual cells.

#### On contractile force derived from the potential energy density of cell edges

The edge contractile force is produced by differentiating the term for edge potential energy in the equations of motion (Eq. S1 in the Supporting materials and methods). Indeed, p-myoII is observed to be enriched on the specific boundary of cells by Ray et al. (18). The boundaries are then expected to become short. However, enrichment of p-myoII on the boundary is not necessarily for the boundary to be shortened. The series of boundaries with p-myoII form polarized myosin supracellular cables and align perpendicularly to the direction of tissue extension (16). Formation of the series of boundaries with p-myoII consisting of unshortened boundaries was also observed in the formation of the neural tube and analyzed using the vertex dynamics (21).

According to the observation of Ray et al. (18), not only p-myoII but also N-cadherin is enriched on specific boundaries of cells. The edge contractile force from the term for edge potential energy is also related to cell-cell adhesion. It may be appropriate that we comment on N-cadherin. When cell-cell adhesion of a cell boundary is strong, the boundary is known to be elongated in the theory of differential cell adhesion in the cell sorting system (25, 26, 27, 28, 29). When cell adhesion is weak, the cell boundary becomes short; that is, the boundary contracts. The observation of N-cadherin on specific boundaries enables us to consider the contribution of N-cadherin to the left-handed helical looping. However, further investigation on the interaction between the two contributions of the contractile molecules and cell-cell adhesion molecules is required.

#### The helical looping process consists of two phases of implicit preparation for looping and explicit remodeling of looping

We demonstrate in Fig. S5 *A* that cell rearrangement took place by frequent reconnections of paired vertices in the early stages of helix looping, but the heart model tube at this stage is almost straight in shape. After this stage, the heart tube is dynamically remodeled, forming the helical loop without cell rearrangement. The helical loop is not made by continuous accumulation of the elemental process of rearrangement but rather by switching from the stage of cell rearrangement to the stage of alteration of the cells themselves. The result may correspond to the proposal by Ray et al. (18) that, before cardiac looping, LR polarization of N-cadherin and myosin II on cell boundaries could lead to LR asymmetric cellular contraction and junctional remodeling.

Furthermore, the mathematical result of the two-phase process may help to explain a discrepancy between two experimental results. Kidokoro et al. (16) reported that when the heart tubes were exposed to the myosin II inhibitors at the HH8/9 stages the treatment blocked the directional extension of cells and the heart tube did not loop (16). Interestingly, heart tubes that were exposed to the myosin II inhibitors at the HH10–12 stages looped normally (6,30,31). In addition, treatment with cytochalasin, which is an inhibitor of actin filament formation, is known to inhibit the looping of the heart tube (6,14). The heart tube at the HH8/9 stage may be implicitly preparing for heart looping, and the heart looping may take place explicitly at the HH10–12 stages by a mechanism that is not inhibited by myosin II inhibitors. However, further detailed investigations of cell rearrangements are required for confirmation.

#### Intrinsic and extrinsic factors causing the left-handed helical looping

The cell chirality of anisotropic cell edge properties that was based on the observation by Ray et al. (18) is the intrinsic factor in the chiral helical looping. We demonstrated the possibility that the intrinsic factor alone, without external factors, determines the handedness of the heart looping. However, Shi et al. (6) have extensive experience with the real heart tube and have noticed the left and right omphalomesenteric veins (OVs) connected caudally to the heart tube. Normally, the left OV is larger and exerts more pushing force than the right OV, causing the heart tube to form with left-handed helical looping. Recently, computer simulations were performed based on detailed observation of developing mouse heart tubes (7). A recapitulation of large-scale dynamic heart looping was demonstrated. In their study, a rightward rotation of the arterial pole and an asymmetric cell ingression in the venous pole were observed. On the basis of the observation of formation of the initial heart tube, Kidokoro et al. (16) described that the left heart cells may more actively rearrange than do right cells, driving asymmetric heart elongation and looping. In our previous study, we introduced the rightward displacement of the anterior part of the heart tube and succeeded in forming the left-handed helical looping (12). The above-mentioned observations belong to extrinsic factors.

We have two questions about the intrinsic and extrinsic factors in the heart tube looping. 1) What is the major cause of the left-handed helical looping, the intrinsic or external factor? 2) Outcomes of the handedness of the helical looping by these two factors seem to be the same. Why are the effects of the two factors consistent? For the first question, we considered that the intrinsic and external factors synergistically determine the handedness of heart looping; that is, an initially subtle asymmetry is amplified via a positive feedback interaction between the intrinsic and external responses. In fact, in this work, we showed that the individual properties of cell chirality caused the left-handed helical looping. On the other hand, the external rightward displacement of the heart model tube has been suggested to cause asymmetrical arrangements of individual cells in the previous study (12). The individual cells and the global deformation of the heart tube may synergistically interact with one another. Both the internal and external factors may not be redundant. For the second question, we think of the nodal signaling pathway, which is known to be a global molecular signaling pathway establishing embryonic laterality of the LR bias. Nodal signaling may be related to the above-mentioned external factors that lead to the left-handed helical looping (the larger left OV, the rightward rotation of the arterial pole, and the LR asymmetric cell ingression in the venous pole). On the other hand, according to Ray et al. (18), myocardial cells constructing the heart tube have CW chirality initially, and nodal signaling reverses the chirality of myocardial cells from CW to CCW. The cells in the right side of the heart tube originating from the nodal-negative lateral plate mesoderm (LPM) exhibit dominant CW chirality, whereas the cells in the left side of the heart tube, receiving contributions from the nodal-positive LPM, exhibit more randomized cellular bias. Ray et al. (18) reported that such a heart tube forms the left-handed helix. When we consider the effect of nodal signaling, we understand that the intrinsic and extrinsic factors consistently work in the heart tube. Desgrange et al. (32) also mentioned that intrinsic and extrinsic mechanisms are not mutually exclusive and may well occur synergistically to drive morphogenesis.

#### Consideration of CE of collective cells across different animal species

Behaviors of myocardial cells in the chick heart have been observed in detail (16). The cells intercalated with each other, and p-myoII was enriched in cell edges aligned along the convergence axis and perpendicularly to the direction of tissue extension, indicating that CE occurred. The myocardial cells in the chick heart tube show asymmetry with rightward-biased edges on which N-cadherin and p-myoII were enriched (18). These data suggest that CE works in the heart tube. Under the assumption of the anisotropic contractile force of edges, we then performed computer simulations using the mathematical model. The computer simulation suggested that CE works in the heart model tube and the heart tube was remodeled into a chiral structure of the left-handed helix. Generally, CE is known to be exerted in axial developmental processes across different animal species (e.g., chick, mouse, *Xenopus,* and zebrafish) (33). We will thus discuss the formation of the chiral heart structure across these animal species.

We have investigated the looping of the mouse embryonic heart (12). Contrary to the heart of the chick embryo, the looping of the mouse embryonic heart is deeply related to the proliferation of cardiomyocytes (12). The mouse heart tube bent in a hairpin fashion through the localized proliferation of cardiomyocytes in the ventral side. By successive anterior-rightward displacement of the tube, we succeeded in remodeling of the bent tube looping to become the left-handed helix. The mechanism of the anterior-rightward displacement of the heart tube had been unclear in the previous work. On the other hand, our results of the chick heart model tube in this study suggest a possibility that anisotropic edge contractile force contributes, through the rightward displacement of the heart tube, to loop formation of the mouse embryonic heart. We thus performed an additional computer simulation. We assumed that the anisotropic edge contractile force works in the mouse heart tube in addition to the cell proliferation; we performed computer simulations as shown in Fig. S8. Fig. S8 *A* is the result of the model tube with only the cell proliferation; the model tube simply bent. When we added an anisotropic contractile force of edges (anisotropic angle, −75°), the model tube became a left-handed helix as shown in Fig. S8 *B*. The ventral view of Fig. S8 *B* shows the anterior-rightward displacement of the tube. Inversely, when the anisotropic angle was +75°, we obtained the right-handed helical loop, as shown in Fig. S8 *C*. It is plausible that the anisotropic contractile force of edges (−75°), via CE of myocardium, caused the anterior-rightward displacement of the mouse heart tube.

The shape of the *Xenopus* heart is a left-handed heart tube similar to chick and mouse hearts (34). CE in the *Xenopus* heart tube, in which CE-defective mutants show heart abnormalities, was experimentally investigated, (33). Because the heart looping of amniotes (birds, mice, and frogs) appears to be similar, there may be a common role for CE in the formation of heart looping.

In contrast to the heart of amniotes, fish do not show a well-defined helix structure of the heart (35, 36, 37, 38, 39), and the mature shape of the fish heart is a flat S shape (32). In the early stage of development of the zebrafish heart tube, the heart tube forms via the fusion of bilateral cardiac cell populations of the LPMs, which are assembled into a disk that rotates clockwise (38). The disk is remodeled to a cone-shaped intermediate, in which ventricular precursors forming the venous end are at the tip and atrial precursors forming the arterial end are at the base. The cone then telescopes out into a tube (40). As the tube undergoes elongation, its venous end is displaced toward the left, accompanying rotation around the axis of the venous portion (38). The ventricular chamber starts to bend rightward, a process referred to as cardiac looping. The outer curvature of the cardiac chambers expands under the constriction at the atrioventricular region. The axis of the looped heart in zebrafish then takes the shape of a flat S. Formation of the zebrafish heart involves rotation of the disk and cone, elongation of the cone, and bending of the cone axis; that is, the CE process is expected to be exerted in the zebrafish heart formation. Indeed, loss-of-function analysis was performed using gene knockdowns, which demonstrates significant impairment of CE (33). In contrast to the normal rightward looping of the heart observed in controls, the heart often failed to loop and instead showed a mirror reversal in the knockdown zebrafish. The CE process seems to contribute to the formation of the chiral structure of the zebrafish heart. Further investigation of the CE is expected to be performed at the cellular level.

## Conclusion

The results of these computer simulations concluded as follows. The anisotropic contractile force of cell edges caused the cell rearrangements, which consequently produced the CE of cell colonies. Cells slipped out of neighboring cells in the convergent region, and cells intercalated between neighboring cells in the extension region. Such deformation took place differently in orientation of the CE between the left and right regions and between the anterior and posterior regions of the heart tube. Thus, the direction of edge contractile force is considered to determine whether the helix loop is left- or right-handed. It should be noted that, despite of the assumption that the distribution of edges with anisotropic contractile force was entirely uniform on the ventral side, the response of colonies in the heart model tube was different in each region. Such regional differences produced the chiral structure of the helical loop.

## Author contributions

H.H. designed the project, performed simulations, and wrote the manuscript.

## Supporting citations

References(41, 42, 43, 44, 45, 46, 47) appear in the Supporting material

## Supporting material

Document S1. Supporting materials and methods, Figs. S1–S8, and Tables S1 and S2

Document S2. Article plus supporting material
