# Supplementary material for: Left-handed cardiac looping by cell chirality is mediated by position-specific convergent extensions
Source: Biophys J. 2021 Oct 23;120(23):5371–83. doi: 10.1016/j.bpj.2021.10.025 (PMC8715179; doi:10.1016/j.bpj.2021.10.025)
Supplement: Document S1. Supporting materials and methods, Figs. S1–S8, and Tables S1 and S2 [file mmc1.pdf]

Biophysical Journal, Volume *120*

Supplemental information

Left-handed cardiac looping by cell chirality is mediated by position-specific convergent extensions

Hisao Honda

Supplemental Information

CONTENTS

—Supplemental Methods

—Figures (Figures S1-S8)

—Tables (Tables S1and S2)

Supplemental Methods

Construction of the initial model tube

We made an initial model tube (diameter of approximately 5.0. Fig. 3 inset) whose lateral surface consisted of 452 polygons (average polygon area was approximately 1) for computer simulations which are described as follows. In a rectangular area (15.75 × 28.8), we distributed 452 circular disks (diameter is 0.82) at random so that the disks did not overlap each other. We used the disk with a diameter of 0.82 to obtain a proper initial distribution. We observed that when the disk diameter was smaller, the distribution of the disks was out of proportion, and when the disk diameter was larger, the amount of time required to obtain the disk distribution was prohibitive. Thus, it was impossible to obtain 452 disks (41). To distribute the disks within the rectangle, the periodic boundary condition was used, that is, the rectangle area under the periodic boundary condition satisfies 2D tiling, in which one side of the rectangle continues to the opposite side of the neighboring rectangle. Then we performed Dirichlet (or Voronoi) tessellation to make a polygonal pattern (42). The rectangle was converted into a cylinder in 3D space as described in Honda et al. (20). We obtained a continuous cell pattern (a seamless tubular cell pattern) on the cylinder side, using the periodic boundary condition. The model tube extremities were adjusted to be the top and bottom circles (strictly speaking, they were polygons consisting of many vertices). The centers of the top and bottom circles were the anterior (cranial or arterial) pole and the posterior (caudal or venous) pole, respectively. The diameter of the initial model tube was approximately 5.0, and each polygonal area in relaxation was 1.0. The model tube was assumed to have belts anteriorly (5% top of the initial model tube) and posteriorly (5% bottom of the initial model tube), where cells were not divided (Fig, S8). The diameters

of the anterior and posterior circles did not change. The polygonal pattern of the side surface of the model tube was expressed by positions of vertices (*x*-, *y*-, *z*-coordinates) of polygons.

Cell polarity

Each polygonal cell was assumed to have its own polarity, which was a reference line of orientations of anisotropic contractile force and cell divisions (Fig. 1A, B, Fig. S1E). However, cell polarities changed during morphogenesis of the model tube because the model tube bent and twisted. We considered that cell polarities were rotated in small amounts around their cell centers during morphogenesis (Fig. S1D). Degrees of small rotations were estimated taking into consideration that cell rotation takes place accompanied by movements of surrounding cells. Using cell A as an example, cell A has n edges and n surrounding cells. Centers of n cells were projected on the plane that includes cell A (Fig. S1D inset). The projected centers were rotated in small amount around the center of cell A in one step of simulation. When their rotation angles were $\Delta\theta_i$ ($i = 1,\ldots, n$), we estimated that the rotation of cell A was $1/n\ \Sigma_i^n\ \Delta\theta_i$. The polarity of cell A was also considered to be rotated by $1/n\ \Sigma_i^n\ \Delta\theta_i$ (dark arrow in Fig. S1D bottom). Such estimations of cell polarities were performed at every step.

Elementary process of reconnecting vertices

While all the vertices moved according to the equations of motion, if an edge between any of the neighboring vertices shrank and reached the small critical length δ, then reconnection of paired vertices of the edge took place as shown in Fig. S1B (43, 44). This process is referred to as the elementary process of reconnecting vertices. Two neighboring polygons designated by * are separated by intercalation of other neighboring polygons, as the topology of the system changes. Since polygons in the process are intercalated with each other, the process brings plastic properties to cell assemblages. We have already indicated that the vertex dynamics involve visco-elastic properties (43). We have introduced a small critical length δ, which defines a minimum length in the model.

Cell-based 3D vertex dynamics

An initial model tube composed of multiple cells is considered as a curved sheet paved with polygons without gaps or overlaps (Fig. S1A). Cells (polygons) in the sheet do not have thickness. The edge (the boundary between two cells) and the area of polygons (cell volume) are expressed as functions with variables of *x, y,* and *z*-coordinates of the vertices. Spatial relationships between neighboring vertices are defined by polygons around the vertices (43, 45).

In this paper, we introduce the system of the vertex model. The vertex model is one which is obtained by coarse-graining real cellular systems in space and time (46). Because the elements of the model are only approximation of real cells, each element has a finite size. We use cell boundary, cell surface and tube volume as the elements. Properties of the elements are statistical averages of the small components of the elements. The finite size defines a minimum length in the model, below which one cannot discuss smaller components. Since a vertex is defined as a point of intersection of cell boundaries, they do not have mass. Vertices receive forces of friction and potential energy. The potential energy in the vertex model involves the energies of the cell boundary, the cell surface and the tube volume, all of which are expressed by the vertices. That is, the motion of the entire system is defined by the vertices. We then assumed that the vertices obey the equation of motion in classical mechanics which is given by

$$\eta \, \mathrm{d}\boldsymbol{r}_i/\mathrm{d}t = -\nabla_i U \qquad (i = 1, \ldots n_\mathrm{v}) \,, \qquad \text{(S1)}$$

where $\boldsymbol{r}_i(t)$ is a 3D-positional vector of vertex i at time t, ∇_i the nabla is differential operator with respect to $\boldsymbol{r}_i$, and n_v is the total vertex number. The coefficient η is a positive constant (an analog of the coefficient of viscosity) and $U(\boldsymbol{r}_i(t))$ denotes the total potential energy. Equation S1 then gives rise to

$$\mathrm{d}U/\mathrm{d}t = \Sigma_\iota \nabla_i U \cdot \mathrm{d}\boldsymbol{r}_i/\mathrm{d}t = -\eta \, \Sigma_\iota (\mathrm{d}\boldsymbol{r}_i/\mathrm{d}t)^2 \leq 0 \qquad \text{(S2)}$$

Equation S2 means the vertices move so that the total potential energy decreases, that is, the mechanical energy is converted into dissipated heat.

The left-hand side of Eq. S1 represents a viscous drag force proportional to the vertex velocity d$\boldsymbol{r}_i$/dt. Vertices do not have mass (inertia) in Eq. S1, so that the motion of the vertices and cells is completely damped. In other words, these vertices are driven

by thermodynamic forces to minimize the total potential energy of the system. This is a slow process of dissipative motions going to an equilibrium, which we examine.

The right side of Eq. S1 represents a potential force (driving force), i.e. minus the gradient of the potential U. The potential U includes various terms related to edges, surface areas of polygons and the tube volume, which are all expressed by vertex positions. Hence, U is a function of the vertex coordinates (x_i, y_i, z_i).

In the present study, the potential U contains terms of edge energy (U_L), elastic surface energy (U_{ES}), elastic volume energy (U_{EV}) and elastic deviation energy of vertices from planes (U_F), and boundary restriction energy of the top and bottom of the tube (U_B):

$$U = U_L + U_{ES} + U_{EV} + U_F + U_B. \qquad \text{(S3)}$$

The potential U_L denotes the total edge (boundary) potential energy of the cells:

$$U_L = \sigma_L \Sigma_{<ij>} w'_{ij} L_{ij}, \qquad \text{(S4)}$$

where i and j are neighboring vertices forming an edge ij, and L_{ij} is length of edge ij. σ_L is edge energy density. w'_{ij} is the summation of weights, w_{ij} of two cells on either side of edge ij. When ($\sigma_L w'_{ij}$) is large, U_L forces edge ij to shorten its edge length L_{ij}, that is, contractile force of edge ij occurs.

The potential U_{ES} denotes the total elastic energy of polygon area:

$$U_{ES} = \kappa_S \Sigma^n{}_\alpha (S_\alpha - S_o)^2, \qquad \text{(S5)}$$

where S_α and S_o are the polygon area at time t and the polygon area at the relaxed state, respectively. κ_S is the elastic energy density of the polygon area. n is the cell number and this increases during the process when cell divisions take place. U_{ES} works so that the area of each polygon becomes the area in the relaxed state.

The potential U_{EV} denotes the elastic energy of the tube volume, where the tube volume is a 3D space that is enclosed by the cylindrical cell sheet and the top and bottom disks:

$$U_{EV} = \kappa_V [V(t) - V_o]^2, \qquad \text{(S6)}$$

where $V(t)$ is the tube volume at time t and V_o is the relaxed state of the tube volume. $V_o(t)$ is a constant at every calculation step, but assumed to be forced to increase in proportion to the calculation step. Calculations at every step were divided into two sub-steps. The adiabatic approximation between the two sub-steps was confirmed to be applicable as follows: (i) Under fixation of V_o (t), the averaged velocity of vertices is

more than δ/h=60, where δ (0.3. See Parameter values) is the minimum length of the system and h is the time interval of the calculation step (0.005). (ii) The tube volume V_o (t) is forced to increase as $V_o(t)$=5.57t + 565: (V_o (t) = 565.0 at t = 0 and V_o (t) = 1400 at t = 150). Since the linear size of the tube can be defined by $L_0(t) = V_0(t)^{1/3}$, the velocity of the change of the linear size of the tube is

$$dL_0(t)/dt = 1/[3V_0(t)^{2/3}]_{t=0}\ dV_0(t)/dt = 5.57/(3\times565^{2/3}) = 0.0272. \qquad \text{(S7)}$$

Then, the velocity of the change of the linear size of the tube is small enough in comparison with the averaged velocity of vertices (> 60) at any time. The adiabatic approximation is justified. The coefficient κ_V is the volume elasticity of the tube. The potential force $-\nabla_i U_{EV}$ in Eq. S1 works so that the total volume of the tube becomes V_o at each calculation step.

The potential U_F denotes the elastic deviation energy of vertices from the planes:

$$U_F = \kappa_F \Sigma_{<j>} (\boldsymbol{r}_j - \boldsymbol{r}_G)^2, \qquad \text{(S8)}$$

where vertex j is connected to three vertices by edges and the three vertices (j_1, j_2, j_3) form a triangle (Fig. S1C). $\boldsymbol{r}_G$ is the center of the triangle, $\boldsymbol{r}_G = (\boldsymbol{r}_{j1} + \boldsymbol{r}_{j2} + \boldsymbol{r}_{j3})/3$. Summations $<j>$ are performed over all vertices except for those that do not belong to the top and bottom polygons. κ_F is the elastic constant of deviation of a vertex from a flat plane. U_F in the equation of motion acts to ensure that the vertices are arranged as flat as possible. The term is necessary in the 3D vertex dynamics of sheets, because polygons in the model do not have thickness. Without the term, neighboring polygons abnormally fold with each other.

The potential U_B denotes boundary restriction energy of top and bottom of the tube:

$$U_B = \kappa_B \Sigma^{nvTop}_j [(\boldsymbol{r}_j - \boldsymbol{r}_{Top})^2 - R_{Top}^2]^2 + \kappa_B \Sigma^{nvBottom}_j [(\boldsymbol{r}_j - \boldsymbol{r}_{Bottom})^2 - R_{Bottom}^2]^2. \qquad \text{(S9)}$$

Centers of the top and bottom polygons (poles) of the tube are $\boldsymbol{r}_{Top}$ and $\boldsymbol{r}_{Bottom}$, respectively. The centers were fixed. The vertices of the top and bottom polygons of the tube (vertex numbers are n_{vTop} and $n_{vBottom}$, respectively) are restricted on the circles of the top and bottom ends of the tube (radius are R_{Top} and R_{Bottom}, respectively). κ_B is the elastic constant of circular array of the vertices of the top and bottom polygons.

Thus, Eq. S1 takes the form:

$$\eta\, d\boldsymbol{r}_i/dt = -\nabla_i [\sigma_L \Sigma^n{}_\alpha (\Sigma^n{}_{\alpha k}\, w'_{ij} L_{\alpha k}) + \kappa_S \Sigma^n{}_\alpha (S_\alpha - S_o)^2 + \kappa_V (V - V_o)^2 + \kappa_F \Sigma^{nv}{}_j (\boldsymbol{r}_j - \boldsymbol{r}_G)^2 + \kappa_B \Sigma^{nvTop}{}_j [(\boldsymbol{r}_j - \boldsymbol{r}_{Top})^2 - R_{Top}^2]^2 + \kappa_B \Sigma^{nvBottom}{}_j [(\boldsymbol{r}_j - \boldsymbol{r}_{Bottom})^2 - R_{Bottom}^2]^2]. \qquad \text{(S10)}$$

In order to remove an explicit parameter corresponding to η, we introduce a new length unit R_o and rewrite Eq. S10 using dimensionless quantities $\boldsymbol{r}_i''$, ∇_i'', S_α'' and V'' as follows:

$$\boldsymbol{r}_i=\boldsymbol{r}_i''R_o,\quad \nabla_i=\nabla_i''/R_o,\quad L_{\alpha k}=L_{\alpha k}''R_o,\quad S_\alpha=S_\alpha'' R_o^{\,2},\quad S_o=S_o''R_o^{\,2},$$
$$V=V''R_o^{\,3},\quad V_o=V_o''R_o^{\,3},\quad \boldsymbol{r}_G=\boldsymbol{r}_G''R_o,\quad \boldsymbol{r}_{Top}=\boldsymbol{r}_{Top}''R_o,$$
$$\boldsymbol{r}_{Bottom}=\boldsymbol{r}_{Bottom}''R_o,\ R_{Top}=R_{Top}''R_o \text{ and } R_{Bottom}=R_{Bottom}''R_o. \qquad \text{(S11)}$$

Thus, Eq. S10 takes the form:

$$\mathrm{d}\boldsymbol{r}_i''/\mathrm{d}t''=-\nabla_i''[\sigma_L''\Sigma^n{}_\alpha(\Sigma^n{}_{\alpha k}\,w'_{ij}L_{\alpha k}'')+\kappa_S''\Sigma^n{}_\alpha(S_\alpha''-S_o'')^2$$
$$+\kappa_V''(V''-V_o'')^2+\kappa_F''\Sigma^{nv}{}_j(\boldsymbol{r}_j''-\boldsymbol{r}_G'')^2+\kappa_B''\Sigma^{nv\mathrm{Top}}{}_j[(\boldsymbol{r}_j''-\boldsymbol{r}_{Top}'')^2-R_{Top}''^{\,2}]^2$$
$$+\kappa_B''\Sigma^{nv\mathrm{Bottom}}{}_j[(\boldsymbol{r}_j''-\boldsymbol{r}_{Bottom}'')^2-R_{Bottom}''^{\,2}]^2]. \qquad \text{(S12)}$$

in which the new quantities are defined as follows:

$$t''=t\sigma_L/(\eta R_o),\quad \sigma_L''=1,\quad \kappa_S''=\kappa_S R_o^3/\sigma_L,\quad \kappa_V''=\kappa_V R_o^5/\sigma_L,$$
$$\kappa_F''=\kappa_F R_o/\sigma_L,\quad \kappa_B''=\kappa_B R_o^{\,3}/\sigma_L. \qquad \text{(S13)}$$

Below, cell motions are measured in terms of the length unit $R_o=S_o^{\,1/2}$ and the time unit $1/(\eta R_o)$. Hereafter, we omit primes (”) on the rescaled quantities in Eq. S12.

$$\mathrm{d}\boldsymbol{r}_i/\mathrm{d}t=-\nabla_i[\Sigma^n{}_\alpha(\Sigma^n{}_{\alpha k}w'_{ij}L_{\alpha k})+\kappa_S\Sigma^n{}_\alpha(S_\alpha-S_o)^2+\kappa_V(V-V_o)^2+\kappa_F\Sigma^{nv}{}_j(\boldsymbol{r}_j-\boldsymbol{r}_G)^2$$
$$+\kappa_B\Sigma^{nv\mathrm{Top}}{}_j[(\boldsymbol{r}_j-\boldsymbol{r}_{Top})^2-R_{Top}^{\,2}]^2+\kappa_B\Sigma^{nv\mathrm{Bottom}}{}_j[(\boldsymbol{r}_j-\boldsymbol{r}_{Bottom})^2-R_{Bottom}^{\,2}]^2]. \qquad \text{(S14)}$$

Parameter values

The system size was defined by $\boldsymbol{r}_{Top}=(28.8, 0, 0)$ and $\boldsymbol{r}_{Bottom}=(0, 0, 0)$, which represent the centers of the top and bottom polygons. The cell number of the tube side at $t = 0$ was $n_C^0 = 452$. When the computer simulation without cell division, the cell number did not change. Cell divisions were assumed in Fig. S8. Polygon area at the relaxed state, $S_o=$ 1.0. Cells of our present model were approximated as a polygon without thickness. The model involves polygons in 3D space, instead of polyhedra. Polygons in 3D space are flat and easily deformed and broken. We sought appropriate values of κ_S, and κ_F. When κ_S was large, each polygon was stable, but hard to be remodeled. When κ_F is small, flat polygons folded easily. To avoid folding of flat polygons, κ_F should be large. We set $\kappa_S= 4$ and $\kappa_F= 1$ for the tube without distinction of the dorsal and ventral sides (Fig. 2) and $\kappa_S= 8$ and $\kappa_F= 1$ for the tube having the specific dorsal side (Fig. 3, Fig. S2 and Fig. S3). Parameters κ_B and κ_V had a wide allowable range and we set $\kappa_B= 0.5$ and $\kappa_V= 0.1$.

We used a large critical edge length $\delta = 0.3$ so that polygons could move and be reformed dynamically in 3D space. Numerical calculations of the differential equations were performed using the Runge-Kutta method with step size $h = 0.005$.

Computer simulations involving cell division

In the case of the model tube involving cell proliferation (Fig. S8), a cell was divided according to its polarity into two daughter cells so that the cell division plane contains the center of the polygon and is perpendicular to the polarity direction as shown in Fig. S1E (red). Cells to be divided were selected at random among cells that were permitted to divide in every small time interval. The interval (t_{interval}) was set 0.4 so that the preceding cell division was not disturbed by the following cell division. Small cells (cell area is less than S_{critical}) were not permitted to divide so that many small polygons did not occupy the tube surface. We set $S_{\mathrm{critical}} = 0.8$. We assumed that the tube volume increased proportionally to the total cell number of the looping heart when cell divisions took place. The assumption was based on data from day 8 to day 11 of mouse embryos (47). The cell number of the tube side at t was n_{C}^{t}. When $t = 0$, n_{C}^{t}. = 452, and $V_{\mathrm{o}} = 1.4\, n_{\mathrm{C}}^{t}$, where V_{o} is fixed during every calculation step. Cells in the anterior-most or posterior-most belt (25% top and bottom of the initial model tube height) were not permitted to divide. $\kappa_{\mathrm{S}} = 8.5$ and $\kappa_{\mathrm{F}} = 1.1$. Positions of the vertices of the top and bottom polygons were fixed.

Centers of sliced model tubes

To analyze shape of a model tube, a model tube in computer simulations was sliced horizontally at levels z_1 and z_2. Maximum and minimum coordinates of cells in the slice were $x_{\max}$, $x_{\min}$, $y_{\max}$ and $y_{\min}$, respectively. We defined $x_{\mathrm{C}} = (x_{\max} + x_{\min})/2$ and $y_{\mathrm{C}} = (y_{\max} + y_{\min})/2$ as the center of the slice. Centers that were vertically stacked were connected to form a chain as shown in Fig. 3B inset. A chain indicates the central tube line. Chains projected on the horizontal plane are shown in the bottom row of Fig. 3B, Fig. S2, Fig. S3, and Fig. S8.

REFERENCES

41. Honda, H., and G. Eguchi. 1980. How much does the cell boundary contract in a monolayered cell sheet? *J. Theor. Biol.* 84:575–588.

42. Honda, H. 1978. Description of cellular patterns by Dirichlet domains: the two-dimensional case. *J. Theor. Biol.* 72:523–543.

43. Honda, H., M. Tanemura, and T. Nagai. 2004. A three-dimensional vertex dynamics cell model of space-filling polyhedra simulating cell behavior in a cell aggregate. *J. Theor. Biol.* 226:439–453.

44. Nagai, T., and H. Honda. 2001. A dynamic cell model for the formation of epithelial tissue. *Philosophical Magazine B.* 81:699–719.

45. Honda, H., and T. Nagai. 2015. Cell models lead to understanding of multi-cellular morphogenesis consisting of successive self-construction of cells. *J. Biochem.* 157:129–136.

46. Nagai, T., S. Ohta, K. Kawasaki, and T. Okuzono. 1990. Computer simulation of cellular pattern growth in two and three dimensions. *Phase Transitions.* 28:177–211.

47. de Boer, B. A., G. van den Berg, P. A. de Boer, A. F. Moorman, and J. M. Ruijter. 2012. Growth of the developing mouse heart: an interactive qualitative and quantitative 3D atlas. *Dev. Biol.* 368:203–213.

Figures

FIGURE S1

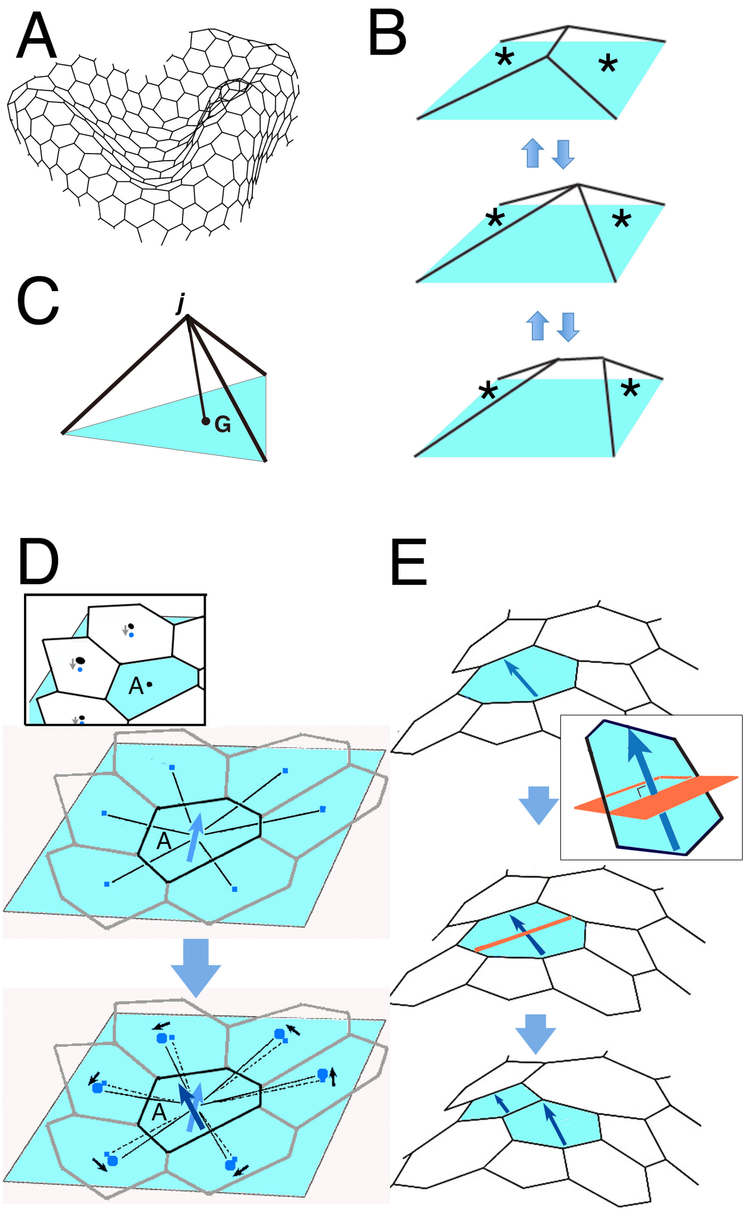

Figures for explanation of the computer simulation

A, A sheet of polygonal pattern in 3D space, which is undulating. Vertices of polygons define shape of a sheet. 3D positional vectors of vertices were calculated using Cell-based 3D vertex dynamics in the Supplemental Methods. Neighbor relationships between vertices of edges define a polygonal pattern. The neighbor relationships change

according to reconnection of paired vertices as shown in *B*. ***B*, **Reconnection of paired vertices of an edge. During reconnection of the vertices, neighboring polygons (identified by *) were separated through intercalation by other neighboring polygons. ***C*,** Deviation of vertex *j* from its triangular plane is expressed by length between *j* and G. Vertex *j* is a tri-junction and has three edges. Three terminals of the edges form a triangular plane. G is the center of the triangle. ***D*,** Small rotation of a cell polarity during morphogenesis. An initial cell polarity (arrow in top figure) rotates (dark blue arrow in bottom figure) according to movements of the peripheral cells (small arrows in bottom figure). For detail see Cell polarity in the Supplemental Methods. *E*, Cell division in 3D space. A cell (blue polygon) has a polarity (blue thin arrow). Cell division (red line) is perpendicular to the polarity. Vertex dynamics remodels the polygonal pattern with a division line (red line) to a new polygonal pattern whose cell number is increased by one. Inset, Cell polarity determines the cell division plane. The cell division plane (red rectangle) is perpendicular to the polarity direction (blue thick arrow) and includes the polygon center.

FIGURE S2

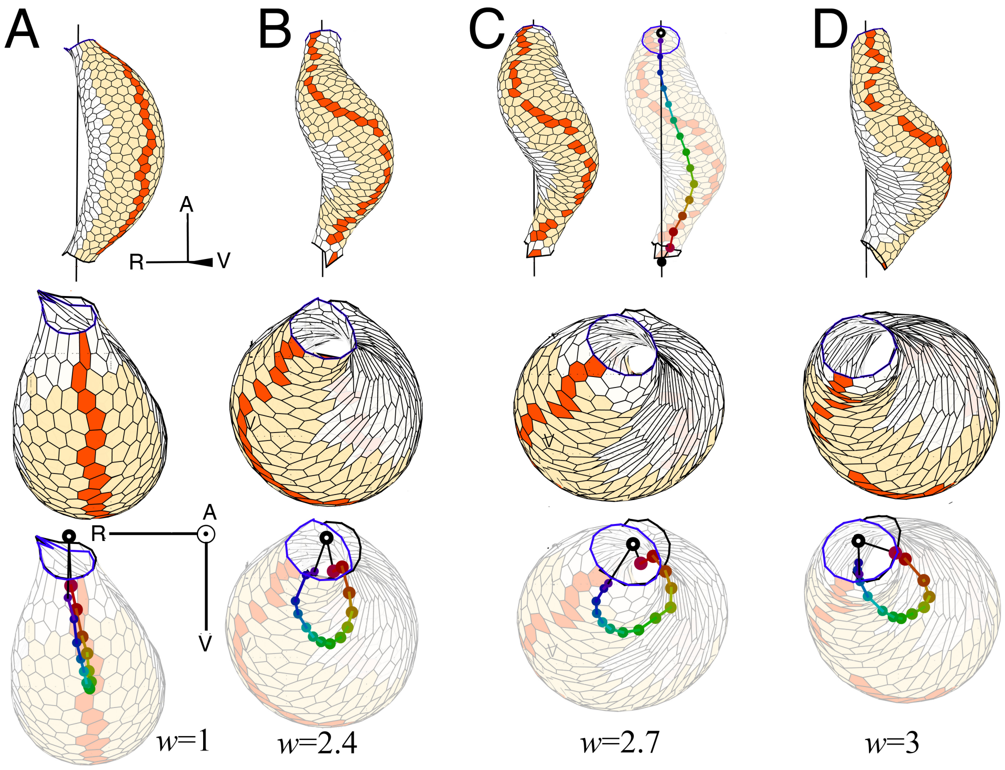

Changes in the shape of the heart model tube depending on the weight of strength of an edge contractile force

The weight of strength of a strong contractile force is *w*, where the weight of strength of a contractile force of other edge is 1. *A–D*, Shapes of the model tube in the computer simulation ($t = 150$) with $w = 1.0$, 2.4, 2.7 and 3.0, respectively. Anisotropic edge angle = −75°. Top and middle rows, side and top views of the model tube. Bottom row, presentation of color chain pattern on which translucent tube image is superimposed. Red polygons are the ventral-most cells in the vertically arranged cell array on the tube surface when $t = 0$. The colored chain indicates the central line of the tube. A, V, and R represent the anterior, ventral, and right directions of the initial model tube, respectively.

FIGURE S3

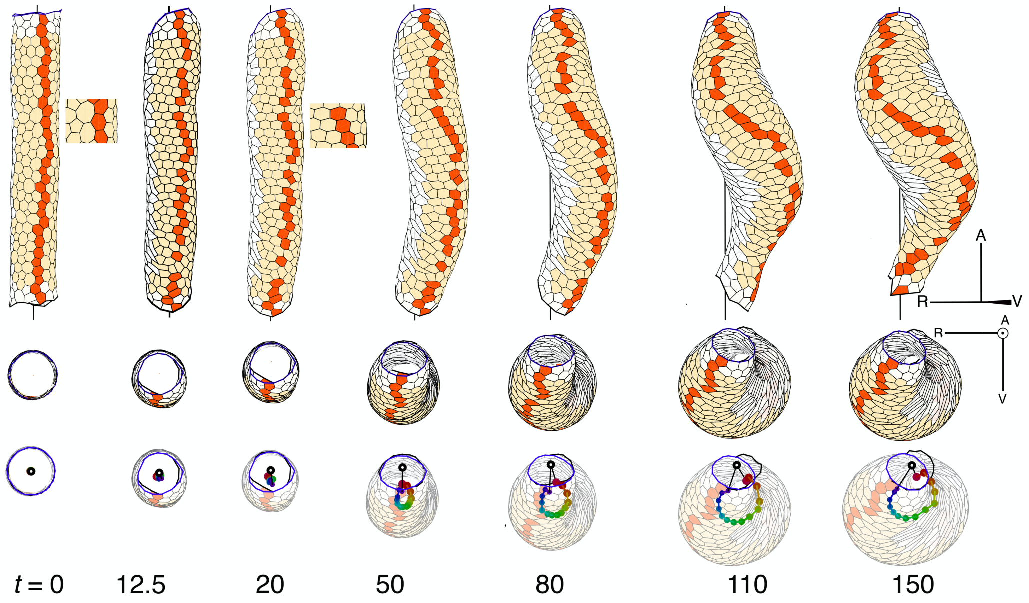

Process of formation of the helical looping of the heart model tube

Anisotropic edge angle = −75°. *w* = 2.7. Top and middle rows, side and top views of the model tube. Bottom row, presentation of color chain pattern on which translucent tube image is superimposed. Inset of *t* = 0 and 20, partially enlarged view. Red polygons are the ventral-most cells in the vertically arranged cell array on the tube surface when *t* = 0. The colored chain indicates the central line of the tube. A, V, and R represent the anterior, ventral, and right directions of the initial model tube, respectively.

FIGURE S4

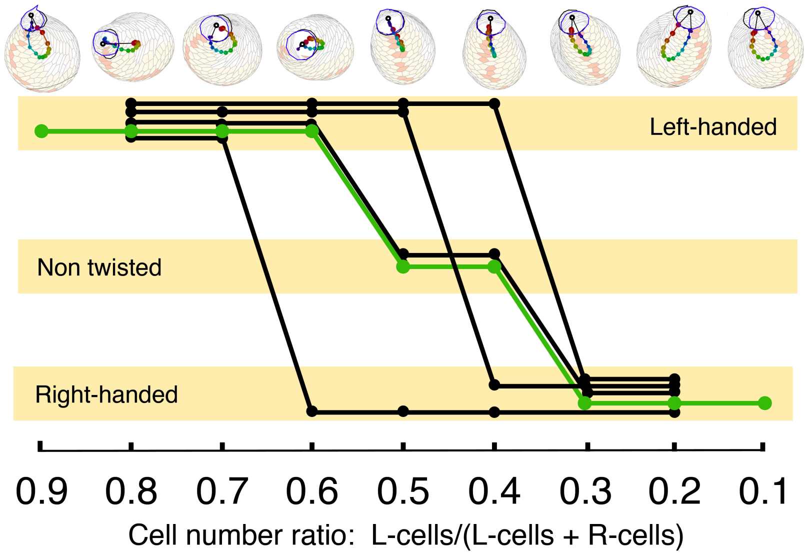

Dependency of the handedness of helical looping on the chirality composition of myocardiac cells in the heart model tube

Using a series of random numbers, model tubes were made with various cell number ratios (L-ratio=L-cell/[R-cell + L-cell]), where L- and R-cells are cells with anisotropic angles −75° and +75°, respectively. Computer simulations of the loop formation were performed and results projected on the horizontal plane are shown (top row). $t = 150$. Bottom figure: Dependence of types of the model tubes (right-handed, non-twisted, or left-handed type) on cell number ratios (L-ratio). Simulations were performed using five different series of random numbers. The green line shows the result of the simulation in top row. Statistical analysis: We obtained five transition ratios at which the handedness change between left and right (0.35; 0.45; 0.45; 0.45; 0.65). Average ± standard deviation (*sd*): 0.47 ± 0.10954. n=5. t-value$_{p=0.05}$ = 2.776 (t-test, degree of freedom 4). Since t-value×$sd/n^{1/2}$ = 0.1360, the confidence interval of the transition ratio with probability 95% is [0.47 – 0.136 ~ 0.47 + 0.136] = [0.334 ~ 0.606]. Therefore, we can say that, when the L-ratio of a model tube is between 0. 606 and 1.0, it will show left-handed helical looping. On the other hand, Ray et al.(*18*) observed six pairs of cell numbers (cell chiralities of CW and CCW). The percentage of CW cells is between 62.5%~74.1% (Table S1). These values of observation are all between 0.606 and 1.0. The results of our computer simulations of the heart model tube are consistent with the observation.

FIGURE S5

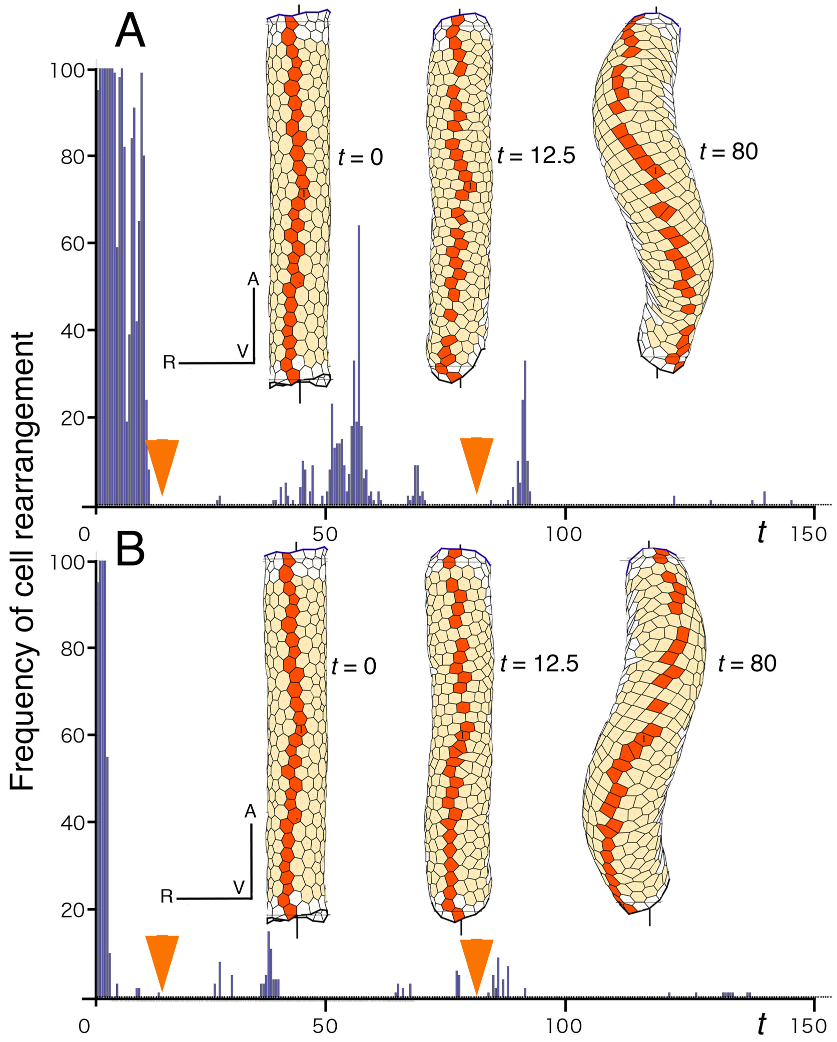

Frequency of cell rearrangement during helical looping of the heart model tube

Frequencies of cell rearrangement per 100 computation steps ($\Delta t = 0.5$) of the computer simulations with a −75° anisotropic angle (*A*) and a +75° anisotropic angle (*B*) are shown. Insets *A* and *B*: Processes of the computer simulations of the left-handed and right-handed helical loop formation under the assumption that the cell rearrangement did not take place after $t = 10$. Orange arrowheads show $t = 12.5$ and 80.

FIGURE S6

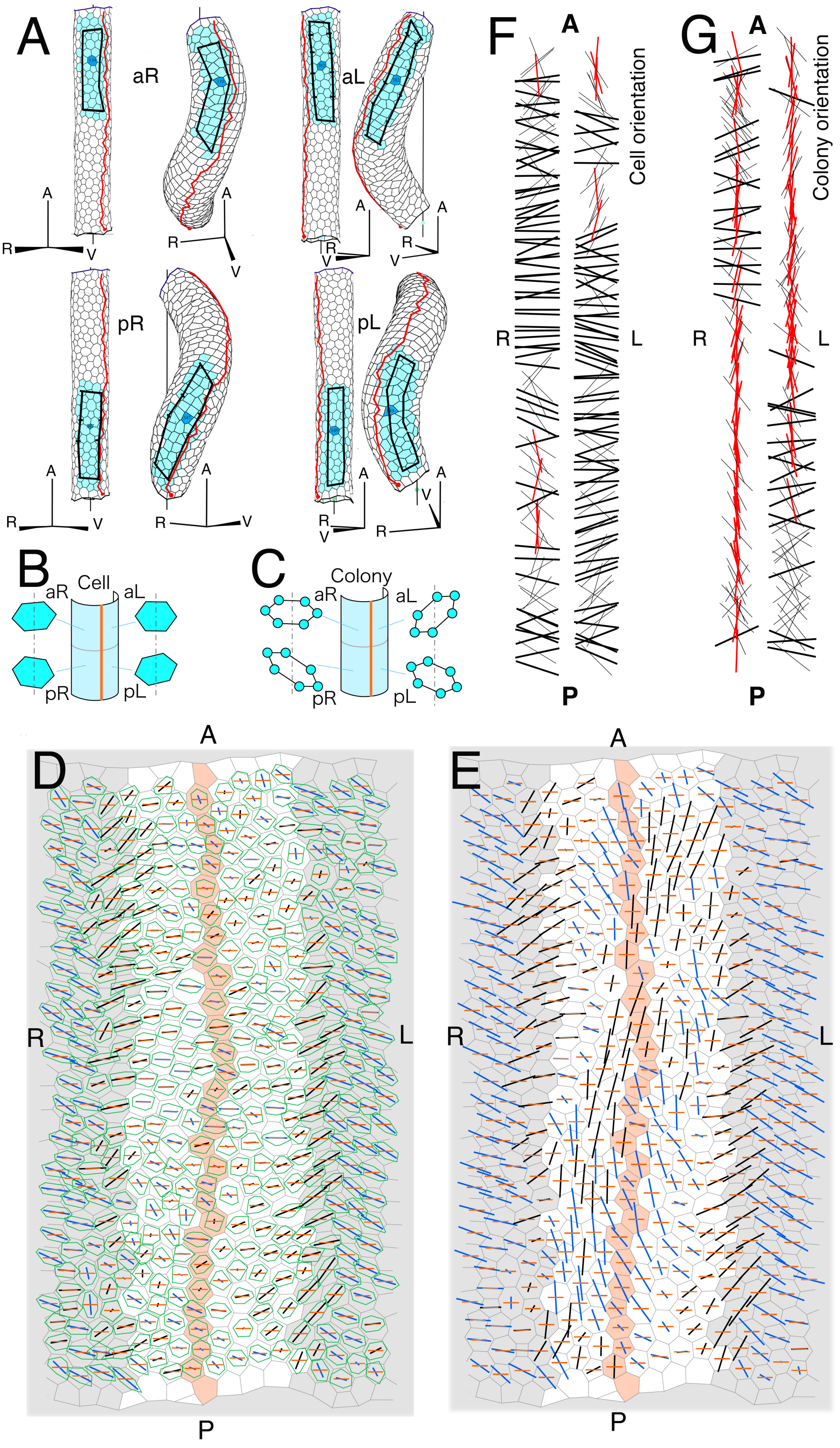

-

Analysis of cell/colony orientations and cell/colony shape anisotropies in the heart model tube with +75° anisotropic angle

A, Regional changes of cell patterns during the helical loop formation of the heart model tube with a +75° anisotropic angle. Cell patterns of the aR, aL, pR and pL regions are shown. Right and left in each figure, heart model tubes at t = 0 and 80, respectively. Cells in each region are faintly colored. Flames of rectangles are drawn for recognition of pattern changes. Each view direction of the four figures is different. The central cell of each region is dark-colored, the normal of which is a view line that is perpendicular to the page. A, R, and V: anterior, right and ventral, respectively. aL, aR, pL, and pR: anterior left, anterior right, posterior left, and posterior right region, respectively. *B, C,* Schematic representation of averaged orientation and shape anisotropy (O_{cell}, A_{cell}) of cells (*B*), and averaged orientation and anisotropy (O_{colony}, A_{colony}) of colonies (*C*) using hexagon, respectively. Averaged orientation and shape anisotropy are expressed by the main axis of the hexagon and the shape of the hexagon, respectively. *D*, Cell orientation (O_{cell}) and cell shape anisotropy (A_{cell}) of a heart tube with a +75° anisotropic angle. Direction and length of line segments indicate the orientation of O_{cell} and relative strength of A_{cell}, respectively. Black and blue lines: angles of line segment from the vertical direction are CW (positive angle) and CCW (negative angle), respectively. Red line indicates the horizontal direction. Green polygon shows a polygonal cell at t = 80 in relative scale. These are plotted on a plane, which is an unfolded sheet of the lateral surface of the initial heart model tube of t = 0. Orange cell array: cells which were at the ventral-most position in the heart tube. Gray zone: dorsal region in the heart model tube. A, P, L, and R: Anterior, posterior, left and right sides, respectively. *E*, Colony orientation (O_{colony}) and colony shape anisotropy (A_{colony}) of a heart tube with a +75° anisotropic angle. Direction and length of line segments indicate the direction of O_{colony} and relative strength of $A_{colony.}$ Other notes, see legend of *D*. *F, G,* Superimposed presentation of line segments of cell orientation and colony orientation. Line segments of cell orientation in the left and right regions of the heart model tube are arranged on each column (*F*). Line segments of colony orientation are arranged similarly (*G*). Lines whose directions are closed to the horizontal line (i.e., absolute angle from the vertical direction, $|O_{cell}| > 60°$, $|O_{colony}| > 60°$) are drawn with thick black line. Lines whose directions are closed to the vertical line (i.e., absolute angle from the vertical direction, $|O_{cell}| < 15°$, $|O_{colony}| < 15°$) are drawn with red line.

FIGURE S7

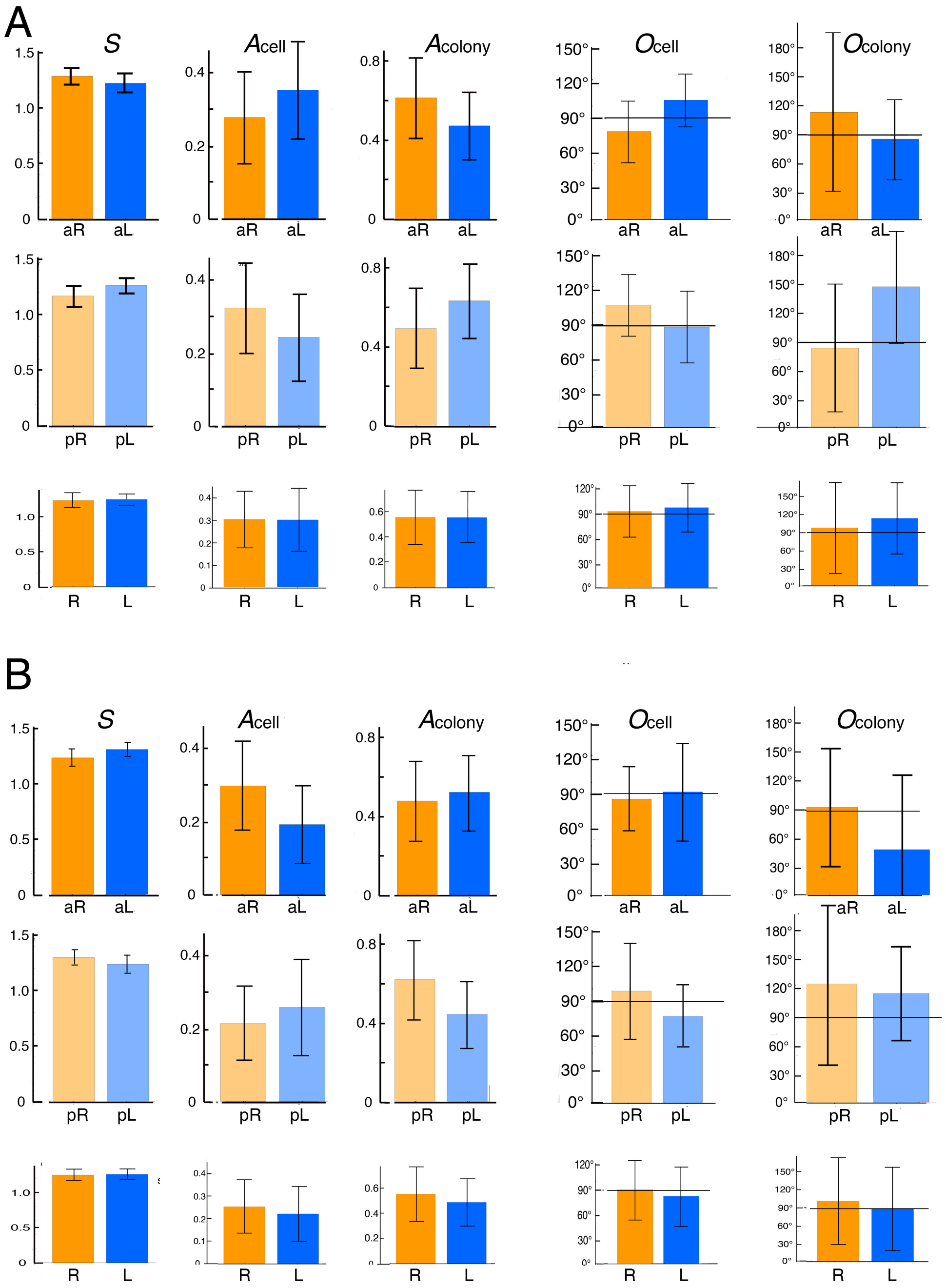

Quantitative data of cell and colony shapes of the heart model tube (Bar chart with standard deviation)

A, B, Model tubes with −75° and +75° anisotropic angles, respectively (See Fig. 5*D, E* and Fig. S6 *D, E*). *S*: cell area. O_{cell}, A_{cell}: cell orientation and cell shape anisotropy, respectively. O_{colony}, A_{colony}: colony orientation and colony shape anisotropy,

respectively. These values are at t = 80. aL, aR, pL, and pR: anterior left, anterior right, posterior left, and posterior right region, respectively. L, R: right and left sides of the heart model tube, respectively.

FIGURE S8

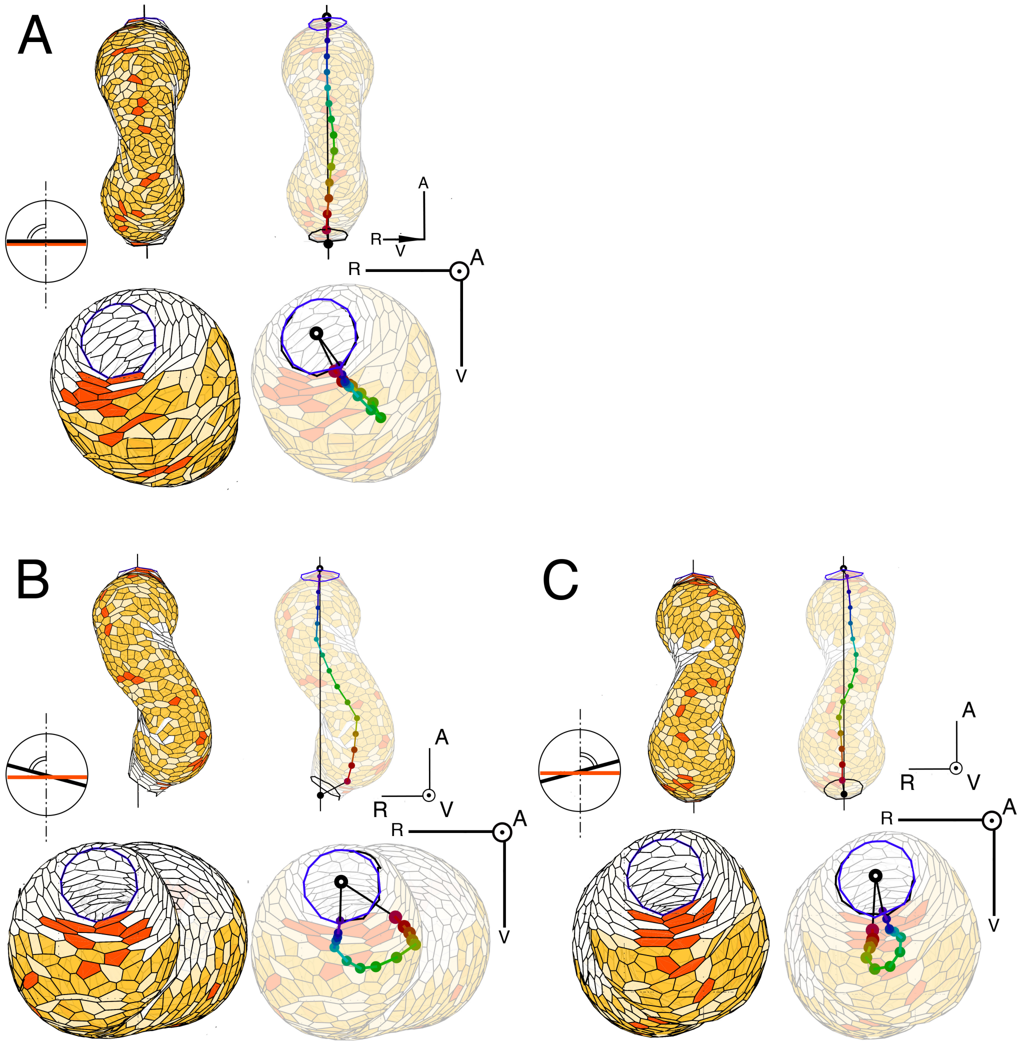

Application of anisotropic contractile force of edges to the model tube of the mouse embryonic heart

The previous computer simulation of the mouse heart model tube involved the assumption of the rightward displacement of the anterior part of the heart model tube (12). Here instead of the rightward displacement of the tube, we assumed the anisotropic contractile force of the edges of the cells. The present mouse heart model tube involved the same cell divisions as the previous model. Ventral cells divided longitudinally between t = 80 and 100 (daughter cells were arranged longitudinally just after division). Division planes were parallel to the horizontal plane as shown by the red

line. An anisotropic edge contractile force was applied with anisotropic angles of −90° (*A*), −75° (*B*) and +75° (*C*). The weight of the strength of a strong edge contractile force, $w = 2.5$. $t = 100$. The bottom shows patterns of colored central chains of the heart model tube (the horizontal view from the top). Color of polygons: faint orange = cells of the ventral half of the initial model tube; orange = cells that were divided during simulation; red = the ventral-most cells at the initial stage. A, R, and V: Anterior, right, and ventral directions of the initial model tube, respectively.

Tables

Table S1 Cell number ratios of CW chirality in Ray et al. (18)

Observation	CW	CCW	Total	%CW	Ray et al. 2018 (18)
1	70	40	110	63.6	Fig.1E HH9
2	35	21	56	62.5	Fig.S3B HH9
3	88	41	129	68.2	Fig.S3D HH9
4	49	25	74	66.2	Fig.S7B HH9
5	55	25	80	68.8	Fig.S7D HH9
6	108	38	146	74.0	Fig.S2 HH9/10
7	125	69	194	64.4	Fig.3I HH9/11
8	108	38	146	74.0	Fig.1E HH10
9	117	41	158	74.1	Fig.2B HH10
10	78	41	119	66.5	Fig.1E HH11
Total	833	379	1212	68.7	

Table S2 Statistics of cell and colony shape of the heart model tube at t = 80

A Anisotropic angle -75°

	Reagion	AV	SD	N	*t*-value: aR-aL / pR-pL	*t*-value: aR-pR	*t*-value: aL-pL
S	aR	1.290	0.074	53	4.076	7.023	
	aL	1.228	0.084	53	*	*	-2.753
	pR	1.174	0.095	52	-5.845		*
	pL	1.269	0.070	52	*		
A_cell	aR	0.277	0.126	53	-2.986	-1.890	
	aL	0.352	0.134	53	*		4.425
	pR	0.323	0.124	52	3.556		*
	pL	0.243	0.119	52	*		
A_colony	aR	0.614	0.205	53	3.855	3.024	
	aL	0.473	0.172	53	*	*	-4.590
	pR	0.494	0.205	52	-3.655		*
	pL	0.635	0.189	52	*		
O_cell	aR	77.311	26.811	53	-5.632	-5.706	
	aL	104.906	23.523	53	*	*	3.086
	pR	107.571	27.482	52	3.351		*
	pL	88.128	31.537	52	*		
O_colony	aR	114.337	83.557	53	2.216	2.024	
	aL	85.827	42.286	53	*	*	-6.360
	pR	84.201	68.390	52	-5.219		*
	pL	149.469	58.758	52	*		
S	R	1.233	0.103	105	-1.232		
	L	1.248	0.080	105			
A_cell	R	0.300	0.127	105	0.0956		
	L	0.298	0.138	105			
A_colony	R	0.555	0.214	105	0.0567		
	L	0.553	0.198	105			
O_cell	R	92.251	31.120	105	-1.116		
	L	96.881	28.960	105			
O_colony	R	96.734	77.221	105	-1.688		
	L	112.844	60.050	105			

B Anisotropic angle +75°

	Reagion	AV	SD	N	*t*-value: aR-aL / pR-pL	*t*-value: aR-pR	*t*-value: aL-pL
S	aR	1.222	0.078	53	-5.357	-5.051	
	aL	1.297	0.064	53	*	*	4.330
	pR	1.294	0.068	52	4.064		*
	pL	1.234	0.081	52	*		
A_cell	aR	0.296	0.120	53	4.811	3.914	
	aL	0.191	0.105	53	*	*	-2.813
	pR	0.212	0.101	52	-1.937		*
	pL	0.256	0.130	52			
A_colony	aR	0.480	0.203	53	-1.157	-3.572	
	aL	0.524	0.194	53		*	2.209
	pR	0.622	0.205	52	4.768		*
	pL	0.445	0.171	52	*		
O_cell	aR	84.612	28.399	53	-0.863	-1.95	
	aL	90.761	43.412	53			2.0105
	pR	98.531	43.090	52	3.1		*
	pL	76.483	27.815	52	*		
O_colony	aR	91.961	61.836	53	3.1982	-2.201	
	aL	47.684	79.590	53	*	*	-5.156
	pR	123.792	84.400	52	0.7295		*
	pL	113.935	48.687	52			
S	R	1.258	0.082	105	-0.711		
	L	1.266	0.080	105			
A_cell	R	0.255	0.119	105	1.8785		
	L	0.223	0.122	105			
A_colony	R	0.550	0.216	105	2.3242		
	L	0.485	0.187	105	*		
O_cell	R	90.947	36.795	105	1.5462		
	L	83.084	36.902	105			
O_colony	R	103.717	74.110	105	1.2326		
	L	91.320	71.621	105			

O_{cell}, A_{cell}: cell orientation and cell shape anisotropy, respectively; O_{colony}, A_{colony}: colony orientation and colony shape anisotropy, respectively; AV*,* SD*,* and N: average, standard deviation, and sample size, respectively; aL, aR, pL, and pR: anterior left, anterior right, posterior left, and posterior right region, respectively; L, R: right and left sides of the heart model tube, respectively. These values are at t = 80; *, Significant p < 0.05. $t_{0.05}$ = 1.982 (degree of freedom =100). See also Fig. 5*D, E* and Fig. S6 *D, E*.
